# Heat Shock Proteins in Cancer: Mechanisms and Therapeutic Targeting

**DOI:** 10.1002/mco2.70966

**Published:** 2026-09-11

**Authors:** Sheng Ma, Chen‐Qian Liu, Si‐Yang Ma, Shao‐Gang Wang, Qi‐Dong Xia, Meng‐Yao Xu

**Affiliations:** ^1^ Department of Urology Tongji Hospital Tongji Medical College Huazhong University of Science and Technology Wuhan China; ^2^ Hubei Provincial Clinical Research Center for Minimally Invasive Treatment of Urology Wuhan China; ^3^ Laboratory of Signaling and Gene Regulation Cecil H. and Ida Green Center for Reproductive Biology Sciences University of Texas Southwestern Medical Center Dallas Texas USA; ^4^ Department of Nuclear Medicine Tongji Hospital Tongji Medical College Huazhong University of Science and Technology Wuhan China

**Keywords:** heat shock proteins, metabolic reprogramming, molecular chaperones, targeted therapy, therapy resistance, precision oncology

## Abstract

Heat shock proteins (HSPs) are molecular chaperones that couple proteostasis to cancer metabolic reprogramming under oncogenic and microenvironmental stress. This Review integrates structural, biochemical, preclinical, and clinical evidence on HSP90, HSP70, HSP60, HSP40, HSP110, and small HSPs. We explain how ATPase cycles, chaperonin cages, co‐chaperone networks, and organelle‐specific localization stabilize metabolic enzymes, glucose transporters, hypoxia‐responsive factors, and electron‐transport‐chain components, thereby coordinating glycolysis, mitochondrial bioenergetics, redox homeostasis, lipid metabolism, and metabolic plasticity. These mechanisms enable tumor survival and therapy resistance but also create context‐dependent vulnerabilities. We critically evaluate pan‐HSP and isoform‐selective inhibitors, disruption of chaperone‐client or co‐chaperone interfaces, HSP‐directed immunotherapies, and biomarker‐guided combinations with metabolic or immune therapies. Clinical translation remains limited by systemic toxicity, compensatory heat‐shock responses, tumor‐type heterogeneity, and the absence of validated predictive biomarkers. We also assess circulating HSPs, cryo‐EM analysis of chaperone complexes, and AI‐assisted ligand design as routes for biomarker development and selective drug discovery. By linking chaperone architecture and client specificity to metabolic outputs and cancer phenotypes, this Review establishes a framework for differentiating actionable HSP dependencies from observational associations. It further defines priorities for patient stratification and precision oncology, supporting the development of selective and tractable strategies that target proteostasis–metabolism coupling.

## Graphical abstract

This review illustrates heat shock proteins (HSPs) as central regulators of cancer metabolic reprogramming. By stabilizing key metabolic enzymes and coordinating glycolysis, oxidative phosphorylation, redox homeostasis, and stress adaptation, HSPs support tumor growth, metastasis, and therapeutic resistance. Targeting HSP networks may therefore provide new opportunities for precision cancer therapy.

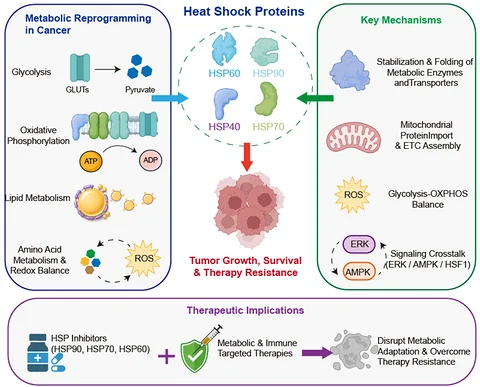

## 1 Introduction

Metabolic reprogramming is a defining feature of cancer that enables malignant cells to meet increased demands for energy, biosynthetic precursors, and redox balance under conditions such as hypoxia, nutrient limitation, and therapeutic stress. The observation that cancer cells preferentially convert glucose to lactate despite adequate oxygen availability established the Warburg effect as an early framework for understanding tumor metabolism. Contemporary models, however, extend beyond aerobic glycolysis to encompass pentose phosphate pathway activation, glutamine utilization, tricarboxylic acid cycle remodeling, mitochondrial plasticity, lipid and cholesterol synthesis, amino acid metabolism, and altered redox control. These interconnected adaptations are regulated by oncogenic signaling, transcriptional programs, posttranslational modifications, and the stability and localization of metabolic enzymes and transporters [1, 2, 3]. These interconnected adaptations are regulated by oncogenic signaling, transcriptional programs, posttranslational modifications, and the stability and localization of metabolic enzymes and transporters.

The extensive metabolic remodeling of cancer cells creates a parallel requirement for protein quality control. Heat shock proteins (HSPs) are molecular chaperones that preserve proteostasis by promoting protein folding, preventing aggregation, facilitating intracellular trafficking, and coordinating the degradation of irreversibly damaged proteins [4, 5]. In tumors, HSP expression and activity are frequently increased in response to proteotoxic, oxidative, metabolic, and microenvironmental stress. Through their client proteins and co‐chaperone networks, HSPs influence the abundance, conformation, localization, and activity of metabolic enzymes, nutrient transporters, signaling kinases, transcription factors, and mitochondrial proteins. HSP‐dependent proteostasis therefore provides a mechanistic interface between cellular stress adaptation and metabolic plasticity, while its effects remain dependent on HSP isoform, subcellular compartment, tumor type, and client repertoire [6, 7].

Although HSPs have been extensively studied as regulators of cancer signaling and therapeutic resistance, their roles in tumor metabolism have often been discussed separately from their structural and chaperone mechanisms. Existing literature has not fully integrated how ATPase‐driven conformational cycles, substrate‐binding domains, chaperonin cages, co‐chaperone interactions, and organelle‐specific localization generate pathway‐level metabolic effects. Moreover, the substantial preclinical activity of HSP‐directed agents has not consistently translated into durable clinical benefit because of systemic toxicity, compensatory heat‐shock responses, limited isoform selectivity, tumor heterogeneity, and the absence of validated predictive biomarkers. This Review addresses these gaps by establishing a structure–client–metabolism framework that links individual HSP families to glycolysis, mitochondrial bioenergetics, lipid metabolism, redox homeostasis, metabolic plasticity, and therapy resistance. It also critically evaluates pharmacological inhibitors, protein–protein‐interaction disruptors, HSP‐directed immunotherapies, biomarker strategies, and emerging approaches for selective therapeutic targeting.

This review first outlines the structural organization, chaperone cycles, subcellular distribution, and functional specialization of the major HSP families, providing a molecular foundation for understanding their context‐dependent roles in cancer. It then examines how HSP90, HSP70, HSP60, and their associated co‐chaperone networks regulate major metabolic pathways. Particular emphasis is placed on the stabilization and localization of glycolytic enzymes and glucose transporters, the folding and assembly of electron transport chain components, mitochondrial protein import, and the regulation of lipid metabolism, biosynthetic pathways, and redox homeostasis. These mechanisms are integrated into a structure–client–metabolism framework that links HSP domain architecture and client specificity to pathway‐level metabolic changes, metabolic plasticity, therapy resistance, and other cancer‐relevant phenotypes. The Review subsequently evaluates HSP‐targeted therapeutic strategies, including ATP‐competitive and isoform‐selective inhibitors, disruption of chaperone–client and co‐chaperone interactions, and HSP‐directed immunotherapies, with critical consideration of clinical efficacy, systemic toxicity, compensatory heat‐shock responses, and resistance mechanisms. Finally, we discuss the principal challenges and future priorities in the field, including predictive biomarker development using circulating and exosomal HSPs, cryo‐EM‐guided characterization of transient chaperone complexes, AI‐assisted ligand discovery, rational combinations with metabolic and immune therapies, and HSP‐based patient stratification for precision oncology.

## 2 Overview of the Heat Shock Protein Family

### 2.1 Classification and Structural Organization

HSPs comprise structurally and functionally distinct chaperone families whose activities are determined by ATP dependence, domain architecture, co‐chaperone interactions, subcellular localization, and client specificity. These features define how individual HSPs recognize unfolded or metastable proteins, regulate protein folding and trafficking, and support stress adaptation in cancer cells [8]. This section summarizes the molecular organization and principal functions of the major HSP families as a foundation for the pathway‐specific metabolic mechanisms.

HSPs are conventionally classified into the HSP110, HSP90, HSP70, HSP60, HSP40, and small HSP families [8]. The ATP‐dependent HSP110, HSP90, HSP70, and HSP60 systems use nucleotide binding and hydrolysis to drive conformational changes during client recognition, folding, release, or complex assembly. HSP40 proteins function primarily as client‐selecting co‐chaperones for HSP70, whereas small HSPs act as ATP‐independent holdases that prevent irreversible protein aggregation. The representative members, core structural domains, chaperone activities, and cancer‐metabolic relevance of these families are summarized in Table 1.

**TABLE 1:** Taxonomy, structural organization, and cancer‐metabolic relevance of major HSP families.

HSP class/taxonomy	Representative human members	ATP dependence	Core structural domains	Principal chaperone role	Cancer‐metabolism relevance	Selected refs.
HSP110/HSPH family	HSPH1 (HSP105), HYOU1/Grp170, HSPA4, HSPA4L	ATP‐dependent; HSP70 nucleotide‐exchange factor (NEF)	HSP70‐like NBD; extended SBD; lid region; C‐terminal acidic segment	Prevents aggregation and accelerates ADP‐ATP exchange on HSP70, thereby controlling the HSP70 folding/refolding cycle	Indirectly maintains the stability of HSP70‐dependent metabolic clients and stress‐adaptation networks; relevant to proteotoxic stress buffering in metabolically rewired tumors	[10, 48, 50]
HSP90/HSPC family	HSP90AA1 (HSP90α), HSP90AB1 (HSP90β), HSP90B1/GRP94, TRAP1	ATP‐dependent folding machine	NTD ATP‐binding pocket; MD client‐binding/catalytic interface; CTD dimerization and co‐chaperone docking region	Matures metastable kinases, transcription factors, and metabolic enzymes through an ATP‐driven open‐closed conformational cycle	Stabilizes HK2, PKM2, HIF‐1α and kinase nodes that sustain glycolysis; TRAP1 regulates mitochondrial bioenergetics and stress signaling	[12, 52, 53, 88]
HSP70/HSPA family	HSPA1A/B (inducible HSP70), HSPA8/HSC70, HSPA5/GRP78, HSPA9/mortalin/mtHSP70	ATP‐dependent folding/translocation machine	NBD; interdomain linker; SBDβ peptide‐binding domain; SBDα lid	Binds exposed hydrophobic peptides, supports folding/refolding, protein translocation, ER stress control and anti‐apoptotic signaling	Controls glucose‐transporter stability/localization, mitochondrial protein import and ER‐stress‐linked metabolic adaptation; GRP78 and mortalin connect proteostasis with therapy resistance	[14, 20, 54, 55, 57]
HSP60/HSPD family	HSPD1/HSP60 with HSPE1/HSP10	ATP‐dependent chaperonin	Equatorial ATP‐binding domain; intermediate hinge domain; apical substrate‐binding domain; HSP10 lid/chaperonin cage	Encapsulates unfolded polypeptides in a mitochondrial folding chamber; essential for folding a subset of mitochondrial proteins	Supports ETC‐complex assembly, OXPHOS, fatty‐acid oxidation and p53‐associated metabolic signaling; context‐dependent tumor‐promoting or tumor‐suppressive activity	[22, 60, 61, 117, 123]
HSP40/DNAJ family	DNAJA, DNAJB and DNAJC subclasses (>40 human members)	ATP‐independent co‐chaperones that regulate HSP70 ATPase activity	Conserved J‐domain with HPD motif; G/F‐rich region; substrate‐binding and dimerization domains (subclass‐dependent)	Defines client selection for HSP70 and accelerates HSP70 ATP hydrolysis to stabilize client capture	Fine‐tunes HSP70‐dependent handling of metabolic enzymes, mitochondrial‐import substrates and stress‐response clients	[26, 66, 67]
Small HSPs/HSPB family	HSPB1/HSP27, HSPB5/αB‐crystallin, HSPB2‐HSPB10	ATP‐independent holdases	Variable NTD; conserved α‐crystallin domain; flexible C‐terminal extension with IXI/V motif	Forms dynamic oligomers that bind partially unfolded proteins and prevent irreversible aggregation	Buffers ROS‐associated proteotoxic stress; HSP27/HSPB1 modulates NF‐κB signaling, ferroptosis sensitivity, cytoskeletal remodeling and lipid‐droplet‐associated stress adaptation	[28, 30, 31, 32, 39]

Abbreviations: ATP, adenosine triphosphate; CTD, C‐terminal domain; ETC, electron transport chain; HIF‐1α, hypoxia‐inducible factor 1α; HK2, hexokinase 2; HSP, heat shock protein; MD, middle domain; NBD, nucleotide‐binding domain; NEF, nucleotide‐exchange factor; NTD, N‐terminal domain; OXPHOS, oxidative phosphorylation; PKM2, pyruvate kinase M2; ROS, reactive oxygen species; SBD, substrate‐binding domain; TRAP1, TNF receptor‐associated protein 1.

### 2.2 The Constituents of the Heat Shock Protein family: Structural Diversity and Functional Specialization

HSPs comprise a highly conserved family of molecular chaperones that are ubiquitously expressed across all domains of life. Classified by their molecular weight, HSPs are traditionally categorized into six major families: HSP110 (∼110 kDa), HSP90 (∼90 kDa), HSP70 (∼70 kDa), HSP60 (∼60 kDa), HSP40 (∼40 kDa), and the small heat shock proteins (sHSPs, 12–43 kDa). Beyond this size‐based classification, a more functionally relevant categorization distinguishes HSPs by their ATP‐dependence. While HSP90, HSP70, HSP60, and HSP110 are ATP‐dependent chaperones that utilize nucleotide binding and hydrolysis to drive allosteric conformational changes essential for substrate folding, sHSPs operate in an ATP‐independent manner, functioning as “holdases” that bind partially unfolded proteins to prevent irreversible aggregation [9]. Critically, the distinct structural domains of these HSPs are not merely architectural features but are fundamental to their mechanistic roles in binding, folding, and stabilizing the client proteins that drive tumor metabolic reprogramming. Table 1 provides a comprehensive summary of the molecular weights, structural characteristics, and functional features of each HSP family.

The HSP110 family (approximately 110 kDa) comprises the largest canonical HSPs. Structurally, HSP110 consists of an N‐terminal nucleotide‐binding domain (NBD) and a C‐terminal substrate‐binding domain (SBD). This architecture enables HSP110 to function either independently as a chaperone that prevents protein aggregation or as a critical nucleotide exchange factor for the HSP70 system [10]. By facilitating the exchange of ADP for ATP on HSP70, HSP110 plays an essential regulatory role in the HSP70 chaperone cycle, thereby indirectly controlling the stability and function of numerous metabolic client proteins.

The HSP90 family (approximately 90 kDa) is one of the most abundant chaperones in eukaryotic cells, comprising four isoforms in mammals: cytosolic HSP90α and HSP90β, endoplasmic reticulum‐localized GRP94, and mitochondrial TRAP1. Structurally, each HSP90 monomer is composed of three highly conserved domains: an N‐terminal domain (NTD) that harbors the ATP‐binding pocket, a middle domain (MD) critical for client protein binding and maturation, and a C‐terminal domain (CTD) that mediates homodimerization and co‐chaperone recruitment [11, 12]. The ATP‐driven conformational cycle of HSP90—alternating between an open “V‐shaped” dimer and a closed, twisted state—is exquisitely tuned for its chaperone function. This cycle directly underpins HSP90's ability to stabilize a repertoire of metastable client proteins, including numerous kinases and transcription factors, as well as metabolic enzymes such as HK2 and PKM2 [12, 13]. By maintaining these clients in an activated, folding‐competent state, HSP90 exerts direct control over glycolytic flux and other metabolic pathways in cancer cells.

The HSP70 family represents one of the most highly conserved and functionally diverse chaperone families, with 13 homologs in mammals differentially expressed across cellular compartments (cytoplasm, nucleus, endoplasmic reticulum, and mitochondria) [14]. Structurally, HSP70 comprises an NBD and a C‐terminal SBD connected by a flexible linker. Allosteric communication between the NBD and SBD—governed by ATP binding and hydrolysis—allows HSP70 to bind and release client peptides with high spatial and temporal precision [15]. Among the most extensively studied members is HSC70 (HSPA8), the constitutively expressed isoform that plays fundamental roles in clathrin‐mediated endocytosis, protein folding, and lysosomal degradation pathways. In contrast, stress‐inducible HSP70 (HSPA1A) is rapidly upregulated in response to proteotoxic stress and is frequently overexpressed in tumors, where it promotes survival and therapeutic resistance [16, 17]. Additionally, GRP78 (HSPA5), an endoplasmic reticulum‐localized HSP70 family member, is a master regulator of the unfolded protein response (UPR) and has been implicated in tumor progression, metastasis, and chemoresistance [18, 19]. The functional diversity of the HSP70 family is further expanded by its extensive network of co‐chaperones, including HSP40 family members and nucleotide exchange factors (NEFs), which dictate substrate specificity and regulate chaperone cycle kinetics [20, 21].

The HSP60 family (approximately 60 kDa) functions as a chaperonin—a large, barrel‐shaped complex that provides a sequestered environment for protein folding. The mammalian HSP60 assembles with its obligatory co‐chaperone HSP10 into a tetradecameric complex comprising two stacked heptameric rings [22]. Each HSP60 monomer contains three distinct structural domains: the equatorial domain (containing the ATP‐binding site), the intermediate domain, and the apical domain (responsible for substrate binding). ATP binding and hydrolysis drive conformational changes that alternately expose the hydrophobic binding surface in the apical domain to capture unfolded polypeptides and then close the chamber to allow folding to occur in an isolated environment [23]. This chaperonin system is essential for the proper folding of approximately 15%–20% of mitochondrial matrix proteins, including subunits of the ETC complexes I, III, and IV, thereby providing a direct structural rationale for HSP60's critical role in regulating OXPHOS in cancer cells [24, 25].

The HSP40 family (approximately 40 kDa) constitutes the largest and most diverse subgroup among heat shock proteins, with over 40 members in humans classified into three subclasses (DNAJA, DNAJB, and DNAJC) based on domain architecture. Structurally, HSP40 contains four conserved domains: (1) the highly conserved J‐domain responsible for interaction with HSP70, (2) a glycine/phenylalanine‐rich (G/F) region, (3) a C‐terminal SBD, and (4) a dimerization domain essential for its chaperone function [26, 27]. Through its J‐domain, HSP40 acts as a critical co‐chaperone that stimulates the ATPase activity of HSP70 and delivers specific client proteins, including metabolic enzymes, to the HSP70 chaperone machine, thereby fine‐tuning the system's activity on distinct metabolic pathways [26].

sHSP family comprises a diverse group of ATP‐independent chaperones ranging from 12 to 43 kDa, with 10 members in humans (HSPB1–HSPB10). Their characteristic structure consists of: (i) a variable NTD that regulates oligomerization and substrate binding, (ii) a conserved α‐crystallin domain that forms the core structure and facilitates dynamic oligomer assembly, and (iii) a flexible C‐terminal extension containing a conserved “IXI/V” motif that contributes to solubility and substrate interactions [28, 29]. Unlike ATP‐dependent chaperones, sHSPs do not actively fold client proteins but instead bind to partially unfolded intermediates, forming stable complexes that prevent irreversible aggregation. This “holdase” activity serves as a first line of defense against proteotoxic stress induced by metabolic imbalances, such as elevated reactive oxygen species (ROS) in tumors [30, 31].

Among the sHSP family, HSP27 (HSPB1) is the most extensively characterized in cancer biology. HSP27 functions as a dynamic oligomer that modulates the stability of client proteins involved in apoptosis, actin polymerization, and the oxidative stress response [31, 32, 33, 34]. αB‐crystallin (HSPB5), another well‐studied member, plays context‐dependent roles in cancer, functioning as either a tumor suppressor or promoter depending on the cellular context and posttranslational modifications [35, 36]. Other family members, including HSPB2–HSPB4 and HSPB6–HSPB10, have been increasingly implicated in various cancers, where they regulate processes such as cell migration, epithelial–mesenchymal transition, and chemoresistance [37, 38, 39]. Notably, sHSPs have emerged as important regulators of metabolic adaptation in tumors, influencing lipid droplet dynamics, redox balance, and mitochondrial function through their ATP‐independent chaperone activity [40].

The structural and functional diversity of HSP families—ranging from ATP‐driven folding machines (HSP90, HSP70, HSP60) to ATP‐independent holdases (sHSPs)—collectively establishes a sophisticated proteostasis network that cancer cells exploit to maintain the stability and activity of metabolic enzymes under stressful microenvironmental conditions. Understanding the distinct structural features of each HSP family is therefore essential for deciphering their specific contributions to tumor metabolic reprogramming and for developing targeted therapeutic strategies.

### 2.3 Biological Functions of Heat Shock Proteins

HSPs are ubiquitously expressed molecular chaperones that play essential roles in maintaining cellular proteostasis. Contrary to the notion that HSPs are primarily localized in mitochondria, different HSP family members exhibit distinct subcellular distributions: HSP90 and HSP70 are predominantly cytoplasmic and nuclear, HSP60 is mitochondrial, GRP78 is endoplasmic reticulum‐resident, and others have specialized localizations [41]. HSPs facilitate a wide range of protein homeostasis functions, including de novo folding of nascent polypeptides, refolding of misfolded proteins, prevention of protein aggregation, targeted degradation of terminally damaged proteins, and regulation of signal transduction complexes [42]. Remarkably, HSPs modulate the functional state of client proteins without altering their primary structures, earning them the designation of “molecular chaperones”.

Under stress conditions such as hyperthermia, hypoxia, oxidative stress, or nutrient deprivation—all commonly encountered in the tumor microenvironment—HSPs are selectively upregulated as part of the cellular stress response, facilitating homeostasis restoration and stress resistance [43, 44]. Their elevated expression in tumor cells enables protective mechanisms that support survival under adverse conditions and contribute to therapy resistance [5, 45]. In the context of cancer metabolism, HSPs exert regulatory control over key metabolic enzymes and signaling hubs, as detailed in subsequent sections [46, 47].

The HSP110 family functions primarily as a protein disaggregase and as the predominant nucleotide exchange factor for cytoplasmic HSP70 [48]. HSP110's NEF activity enhances ADP/ATP exchange on HSP70, thereby accelerating the HSP70 chaperone cycle. Through this mechanism, HSP110 participates in nearly all HSP70‐associated processes, including stress‐induced de novo folding and refolding of newly synthesized or damaged proteins, dissolution of protein aggregates, and targeted proteolysis [10, 49]. Importantly, by modulating HSP70 activity, HSP110 indirectly influences the stability of metabolic enzymes that are clients of the HSP70 system, such as key glycolytic enzymes and mitochondrial import substrates [50, 51].

The HSP90 family (comprising cytosolic HSP90α and HSP90β, ER‐resident GRP94, and mitochondrial TRAP1) maintains the stability and functional maturation of a distinct set of client proteins, the majority of which are involved in signal transduction, including protein kinases (AKT, RAF, HER2), transcription factors (HIF‐1α, p53), and steroid hormone receptors [11]. Among the cytosolic isoforms, HSP90α is preferentially heat‐inducible and plays crucial roles in tumor cell proliferation and stress adaptation, whereas HSP90β is constitutively expressed, mitotically induced, and associated with cellular differentiation and cytoskeletal organization [52]. The mechanistic link between HSP90 and cancer metabolism is direct: HSP90 stabilizes key metabolic enzymes such as HK2 and PKM2, thereby promoting glycolytic flux in cancer cells [53]. Additionally, HSP90 facilitates the maturation of HIF‐1α, a master transcriptional regulator of metabolic reprogramming that induces the expression of glucose transporters (GLUT1) and glycolytic enzymes [12]. Thus, HSP90 serves as a central node integrating oncogenic signaling with metabolic adaptation.

The HSP70 family (13 homologs in humans, including stress‐inducible HSP70/HSPA1A, constitutively expressed HSC70/HSPA8, and ER‐resident GRP78/HSPA5) performs multifaceted functions: (i) facilitating de novo folding of nascent polypeptide chains and refolding of misfolded proteins through an ATP‐dependent cycle; (ii) assisting protein translocation across mitochondrial and endoplasmic reticulum membranes; and (iii) conferring apoptosis resistance by sequestering pro‐apoptotic factors such as Bax and Apaf‐1) [20, 54]. In the context of metabolism, HSP70 interacts with and stabilizes glucose transporter GLUT1 at the plasma membrane, promoting glucose uptake in cancer cells [55, 56]. Moreover, mitochondrial HSP70 (mtHSP70/mortalin) is essential for the import of nuclear‐encoded mitochondrial proteins, including subunits of the ETC, thereby sustaining OXPHOS [57, 58]. GRP78, as a master regulator of the URP, coordinates metabolic adaptation under ER stress conditions commonly encountered in rapidly proliferating tumors [59].

The HSP60 family, a mitochondrial chaperonin, functions together with its co‐chaperone HSP10 to form a large barrel‐shaped complex that provides an isolated compartment for ATP‐dependent protein folding [60]. Within the mitochondrial matrix, the HSP60/HSP10 complex folds approximately 15%–20% of all mitochondrial proteins, including subunits of the electron transport chain complexes I, III, and IV, as well as key metabolic enzymes such as acyl‐CoA dehydrogenases involved in fatty acid oxidation [61, 62]. Beyond its mitochondrial roles, HSP60 has been detected in the cytosol and extracellular space under pathological conditions. In the cytosol, moderate levels of HSP60 bind and sequester the pro‐apoptotic factor Bax, enhancing stress tolerance, whereas excessive accumulation triggers cytochrome c release and apoptosis [46, 60]. Extracellularly, HSP60 can act as a danger‐associated molecular pattern (DAMP), inducing T‐cell differentiation and pro‐inflammatory cytokine secretion [63, 64]. In cancer, HSP60 exhibits context‐dependent duality: it promotes proliferation and metabolic reprogramming in pancreatic cancer and multiple myeloma, yet suppresses tumorigenesis in clear cell renal carcinoma and hepatocellular carcinoma [65]. This functional duality is likely dictated by cell type‐specific client proteins, posttranslational modifications, and subcellular localization.

The HSP40 family functions as essential co‐chaperones for HSP70, stimulating its ATPase activity and delivering specific client proteins to the HSP70 chaperone machine [66]. Humans express over 40 HSP40 members classified into three subclasses (DNAJA, DNAJB, DNAJC) based on domain architecture [66, 67]. By dictating substrate specificity, HSP40 proteins enable the HSP70 system to act on a diverse array of clients, including metabolic enzymes, thereby fine‐tuning metabolic pathways according to cellular demands.

sHSPs operate in an ATP‐independent manner, functioning as “holdases” that bind partially unfolded proteins to prevent irreversible aggregation. Their dynamic oligomeric assemblies, built around the conserved α‐crystallin domain, allow rapid response to stress conditions. In cancer metabolism, sHSPs play emerging roles: HSP27 (HSPB1) stabilizes lipid droplet‐coating proteins (PLIN1/PLIN2), promoting lipid storage, and also activates the IKK/NF‐κB pathway to enhance survival under oxidative stress [68, 69]. αB‐crystallin (HSPB5) has been implicated in metabolic adaptation and chemoresistance in multiple tumor types [18].

Aberrant HSP expression correlates with numerous pathological conditions, including cancer, neurodegenerative disorders, metabolic diseases, cardiovascular pathologies, autoimmune diseases, and chronic inflammatory conditions [70]. In cancer, HSPs exhibit dualistic roles, functioning either as tumor suppressors or promoters depending on the cellular context, cancer type, and specific family member. For example, while HSP60 serves as a cancer‐associated protein marker in colorectal, pancreatic, and breast cancers, it may conversely exhibit tumor‐suppressive effects in hepatocellular carcinoma and clear cell renal cell carcinoma [71, 72]. Understanding this context dependency is critical for developing HSP‐targeted therapies with acceptable therapeutic windows.

## 3 Mechanisms of Heat Shock Proteins in Regulating Tumor Metabolism

Cancer metabolic reprogramming is sustained by coordinated regulation of nutrient uptake, metabolic‐enzyme stability, organelle proteostasis, and stress‐responsive signaling rather than by alterations in a single pathway. This section examines how HSPs and their co‐chaperone networks regulate tumor metabolism at three interconnected levels: direct stabilization, folding, or trafficking of metabolic client proteins; maintenance of mitochondrial and endoplasmic‐reticulum proteostasis; and modulation of signaling pathways that coordinate metabolic adaptation. The discussion proceeds from glycolysis and glucose uptake to mitochondrial protein import and oxidative phosphorylation, broader bioenergetic regulation, lipid and biosynthetic metabolism, and reactive oxygen species homeostasis. The final subsection integrates these mechanisms into a structure–client–metabolism–phenotype framework, distinguishing direct chaperone‐dependent effects from indirect compensatory responses and explaining how HSP‐regulated proteostasis contributes to metabolic plasticity, stress survival, and therapy resistance.

### 3.1 The Pivotal Role of Heat Shock Proteins in Tumor Metabolism

**FIGURE 1:**
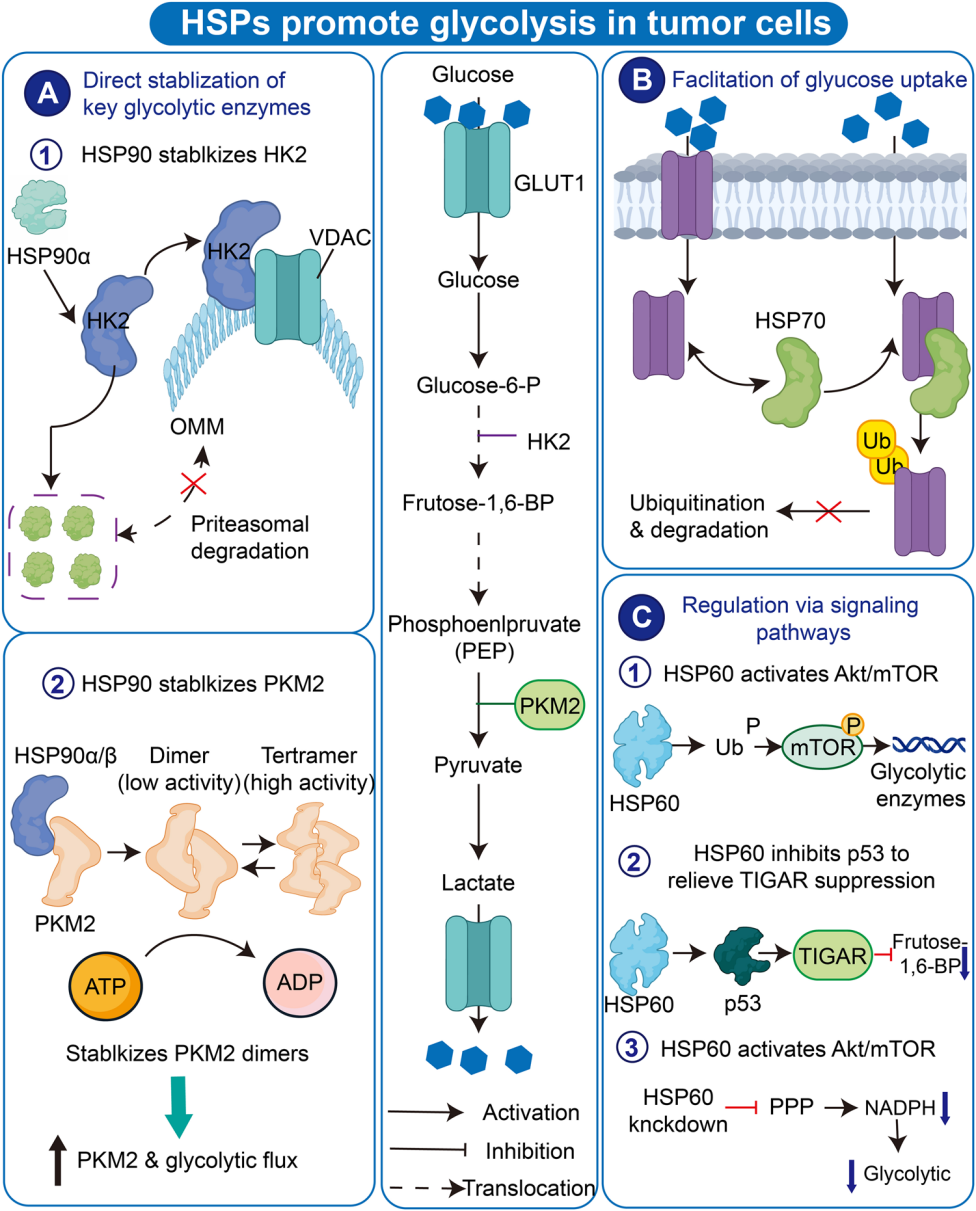
HSP‐mediated regulation of glycolysis in cancer cells. (A) Direct regulation of glycolytic enzymes by HSP90. A1: HSP90α binds and stabilizes HK2 at the outer mitochondrial membrane, thereby limiting proteasomal degradation and supporting the phosphorylation of glucose. A2: HSP90α and HSP90β regulate PKM2 conformation and stability, thereby supporting PKM2‐dependent glycolytic flux. **(B)** HSP70 stabilizes GLUT1 at the plasma membrane and limits ubiquitination‐mediated degradation, thereby sustaining glucose uptake. **(C)** HSP60 regulates glycolysis through stress‐responsive signaling pathways. C1: HSP60 promotes Akt–mTOR signaling and the expression of glycolytic enzymes. C2: HSP60–p53 interactions attenuate p53 activity and relieve TIGAR‐mediated suppression of glycolysis. C3: HSP60 depletion disrupts pentose phosphate pathway activity, decreases NADPH production, and reduces glycolytic and biosynthetic capacity. Regular solid arrows indicate direct molecular events or directional processes; dashed arrows indicate indirect or multistep relationships; blunt‐ended lines indicate inhibition; red crosses indicate blockade of the depicted process; and bold colored arrows indicate the net increase or decrease in metabolic output. GLUT1, glucose transporter 1; HK2, hexokinase 2; HSP, heat shock protein; mTOR, mechanistic target of rapamycin; NADPH, reduced nicotinamide adenine dinucleotide phosphate; OMM, outer mitochondrial membrane; PEP, phosphoenolpyruvate; PKM2, pyruvate kinase M2; PPP, pentose phosphate pathway; TIGAR, TP53‐induced glycolysis and apoptosis regulator; Ub, ubiquitin; VDAC, voltage‐dependent anion channel.

HSPs regulate glycolysis through direct stabilization of rate‐limiting enzymes and transporters, as well as indirect modulation of signaling pathways (Figure 1).

#### 3.1.1 HSPs Modulate the Stability and Function of Key Glycolytic Enzymes

For instance, HSP90 enhances glycolytic flux by directly interacting with rate‐limiting enzymes. HK2, which catalyzes the first committed step of glycolysis by phosphorylating glucose to glucose‐6‐phosphate, is predominantly localized on the outer mitochondrial membrane [73]. Overexpressed in numerous cancers, HK2 drives the Warburg effect [74, 75], and its activity is positively regulated by HSP90. Mechanistically, the cytosolic HSP90α isoform has been identified as the primary chaperone that binds to and stabilizes HK2 on the mitochondrial outer membrane, preventing its degradation and promoting glucose metabolic influx [76]. Similarly, HSP90 regulates PKM2, the final rate‐limiting enzyme in glycolysis that converts phosphoenolpyruvate (PEP) and ADP into pyruvate and ATP. In tumor cells, PKM2 exists in a dynamic equilibrium between highly active tetramers and less active dimers/monomers. The latter can translocate to the nucleus, functioning as a protein kinase to modulate glycolytic gene expression [77]. Studies demonstrate that the ATPase cycle of both HSP90α and HSP90β contributes to stabilizing PKM2 dimers, enhancing their enzymatic activity under high‐glucose conditions [78], thereby supporting tumor glycolytic demands [79]. Thus, HSP90 isoforms exert overlapping yet potentially context‐dependent control over distinct glycolytic enzymes.

#### 3.1.2 HSPs Also Promote Glycolysis by Facilitating Glucose Uptake

HSP70 interacts with glucose transporters, stabilizing their membrane localization and preventing ubiquitin‐mediated degradation. For example, in non–small cell lung cancer (NSCLC), HSP70 expression correlates positively with GLUT1 levels and glucose uptake [80].

Beyond direct interactions, HSPs regulate glycolysis through metabolic signaling cascades. HSP60, for instance, influences mitochondrial function under stress, thereby altering downstream glycolytic pathways. In myeloma cells, HSP60 knockdown disrupts the pentose phosphate pathway, reducing glycolytic and TCA cycle intermediates by impairing ribonucleotide and NADPH synthesis [81]. Moreover, HSP60 activates the Akt/mTOR axis to upregulate glycolytic enzyme transcription, ensuring sustained energy production [82]. Notably, HSP60 modulates glycolysis by interacting with the tumor suppressor p53. The chaperonin cavity of HSP60, formed by its apical domains, can facilitate the proper folding or complex assembly of signaling molecules like p53. By forming complexes with p53, HSP60 attenuates p53 activity, diminishing its suppression of TIGAR (TP53‐induced glycolysis and apoptosis regulator)—a p53 target gene that inhibits glycolysis by reducing fructose‐2,6‐bisphosphate levels—thereby indirectly boosting glycolytic flux [83, 84, 85].

### 3.2 Regulatory Mechanisms of Heat Shock Proteins in Oxidative Phosphorylation

**FIGURE 2:**
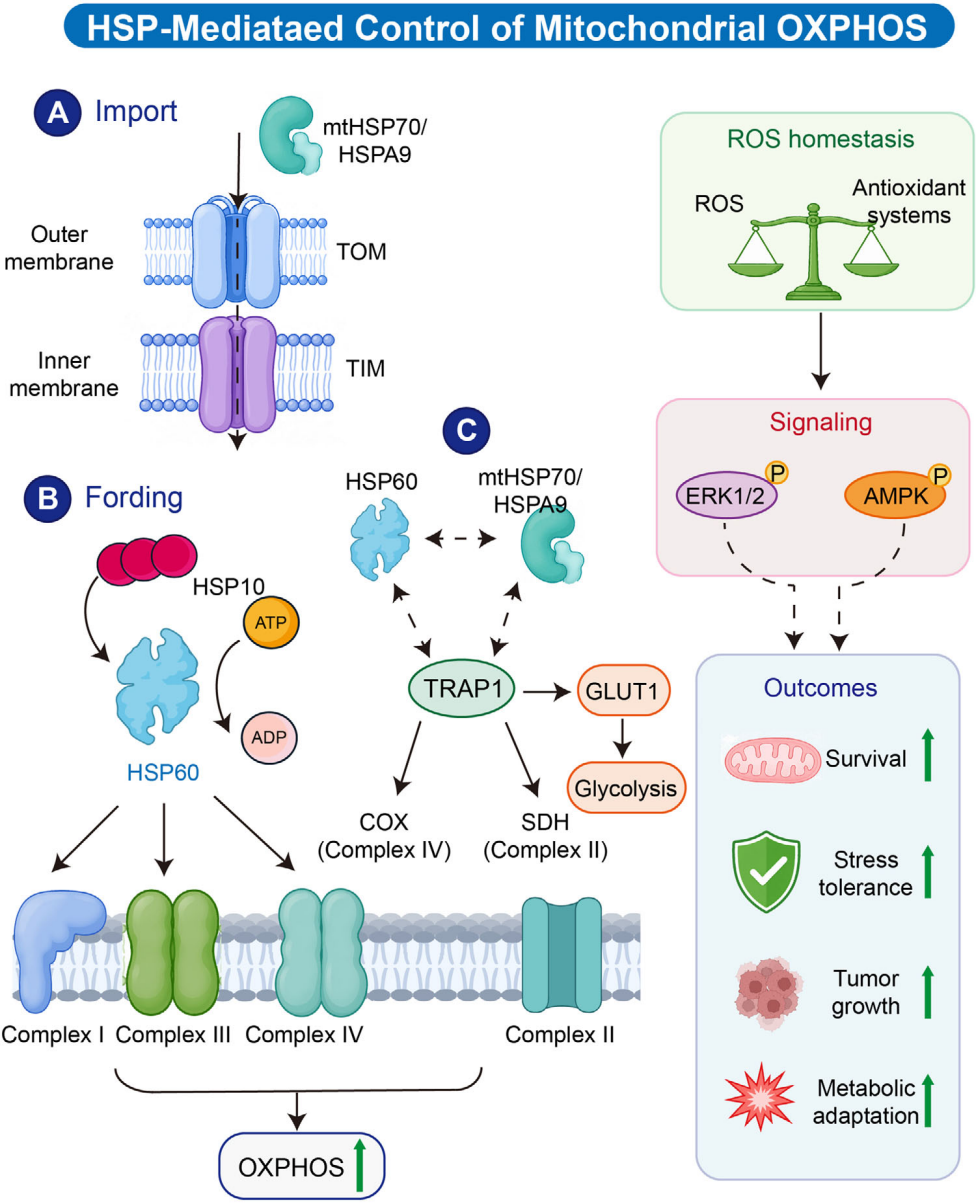
Regulatory mechanisms of heat shock proteins in mitochondrial oxidative phosphorylation. (A) mtHSP70/HSPA9 cooperates with the TOM and TIM translocases to support the import of nuclear‐encoded mitochondrial proteins, including electron‐transport‐chain subunits and metabolic enzymes. (B) The ATP‐dependent HSP60–HSP10 chaperonin system promotes the folding and assembly of mitochondrial proteins required for respiratory complexes I, III, and IV, thereby sustaining oxidative phosphorylation and ATP production. (C) HSP60, mtHSP70, and TRAP1 form an integrated mitochondrial proteostasis network. TRAP1 modulates cytochrome‐c oxidase, succinate dehydrogenase, respiratory output, and GLUT1‐associated glycolytic adaptation, whereas ROS‐dependent ERK1/2 and AMPK signaling connects mitochondrial function to tumor‐cell survival, stress tolerance, growth, and metabolic adaptation. Regular solid arrows indicate direct molecular events or directional processes; dashed arrows indicate indirect, cooperative, or multistep relationships; and bold colored arrows indicate the net increase or decrease in pathway activity or phenotype. AMPK, AMP‐activated protein kinase; COX, cytochrome‐c oxidase; ERK1/2, extracellular signal‐regulated kinases 1 and 2; ETC, electron transport chain; GLUT1, glucose transporter 1; HSP, heat shock protein; mtHSP70, mitochondrial HSP70; OXPHOS, oxidative phosphorylation; ROS, reactive oxygen species; SDH, succinate dehydrogenase; TIM, translocase of the inner mitochondrial membrane; TOM, translocase of the outer mitochondrial membrane; TRAP1, TNF receptor‐associated protein 1.

Mitochondrial OXPHOS is critically dependent on HSPs for the folding, assembly, and import of electron transport chain (ETC) components (Figure 2).

#### 3.2.1 HSP60‐Mediated Regulation of ETC Complex Assembly

As an essential mitochondrial chaperonin, HSP60 plays a critical role in regulating the assembly of mitochondrial complexes during OXPHOS. In conjunction with HSP10, HSP60 forms a folding complex that ensures proper folding of key proteins within the ETC. The ATP‐hydrolysis‐driven conformational changes within HSP60's equatorial and apical domains are essential for encapsulating and folding nascent polypeptide chains of ETC subunits. Studies demonstrate that HSP60 is indispensable for the stability of Complexes I, III, and IV. Deficiency of HSP60 disrupts the assembly of these complexes, leading to significant suppression of ATP production and ultimately impairing cellular energy metabolism [86]. Mechanistically, HSP60‐mediated proper folding of ETC subunits prevents the accumulation of misfolded intermediates that would otherwise generate excessive ROS and trigger mitophagy.

#### 3.2.2 HSP70‐Mediated Mitochondrial Protein Import

The stability of mitochondrial inner membrane proteins relies on the assistance of HSPs. Mitochondrial heat shock protein 70 (mtHSP70/mortalin, encoded by HSPA9), a member of the HSP70 family localized in the mitochondrial matrix, utilizes its canonical SBD and ATP‐driven conformational cycle to collaborate with the mitochondrial protein import machinery (TIM/TOM complex). mtHSP70 binds to precursor polypeptides as they emerge from the TOM complex into the matrix, using ATP hydrolysis to drive translocation and facilitate proper folding [87, 88]. Key classes of proteins imported via this machinery include: (i) subunits of ETC complexes (ND1, COX1, Cyt b), (ii) matrix enzymes involved in the TCA cycle (e.g., citrate synthase, aconitase), (iii) mitochondrial transcription and replication factors, and (iv) chaperones themselves [87, 88]. In glioblastoma, mtHSP70 sustains OXPHOS‐dependent energy supply by activating the TIM/TOM complex to augment mitochondrial protein import [87, 88].

#### 3.2.3 TRAP1 and Metabolic Rewiring

TNF receptor‐associated protein 1 (TRAP1), a mitochondrial isoform of cytosolic HSP90, forms complexes with HSP60 and mtHSP70 to collectively promote metabolic rewiring in tumors. Unlike cytosolic HSP90, TRAP1 localizes to the mitochondrial matrix and intermembrane space, where it regulates the activity of several client proteins involved in energy metabolism. Specifically, TRAP1 modulates the activity of cytochrome c oxidase (COX) and succinate dehydrogenase (SDH), enzymes critically involved in controlling GLUT1 expression [89]. Through GLUT1 modulation, TRAP1 indirectly influences the balance between glycolysis and mitochondrial function, further supporting tumor cell survival and proliferation [90]. Mechanistically, TRAP1 achieves this by stabilizing SDH, thereby preventing succinate accumulation and subsequent HIF‐1α stabilization, which would otherwise promote glycolytic gene expression. Thus, TRAP1 acts as a metabolic rheostat that fine‐tunes the glycolysis‐OXPHOS balance.

#### 3.2.4 HSP60‐Mediated ROS Signaling and Cancer Cell Proliferation

HSP60 exerts a complex, context‐dependent effect on ROS production and cancer cell proliferation. In pancreatic cancer cells, HSP60 upregulates Erk1/2 phosphorylation by enhancing OXPHOS activity, thereby promoting cell growth and proliferation [24]. Conversely, in clear cell renal carcinoma, downregulation of HSP60 induces ROS accumulation, activating the AMP‐activated protein kinase (AMPK) signaling pathway and triggering adaptive cellular responses [65]. This seemingly paradoxical relationship—where both HSP60 gain‐ and loss‐of‐function can promote tumorigenesis under different contexts—is mechanistically resolved by considering the level of ROS produced. Mild to moderate ROS generated upon modest HSP60 dysfunction activates pro‐survival pathways that enhance antioxidant defenses and metabolic adaptation, thereby supporting tumor growth [71, 91]. In contrast, severe HSP60 loss leads to overwhelming ROS production, triggering cell death [92]. The cancer cell therefore maintains HSP60 expression within an optimal range to achieve a “just‐right” ROS level that promotes proliferation without inducing cytotoxicity. This concept of a ROS threshold for tumor promotion versus suppression is critical for understanding HSP60's dualistic role. These findings underscore HSP60's role as a bridging molecule that balances metabolic pathways and coordinates signaling cascades, highlighting its pathophysiological significance.

#### 3.2.5 ERK and AMPK Signaling in HSP‐Mediated Metabolic Control

The signaling cross‐talk between HSPs and key metabolic regulators further integrates cellular energy status with chaperone activity. HSP60 has been shown to activate the ERK1/2 pathway, which in turn promotes mitochondrial biogenesis and OXPHOS gene expression [24]. Conversely, AMPK activation, induced by ROS or energy stress following HSP dysfunction, can feed back to regulate HSP expression and activity [93, 94]. For instance, AMPK directly phosphorylates and activates HSF1, the master transcriptional regulator of HSPs, creating a feed‐forward loop that enhances cellular stress tolerance [94, 95, 96]. The interplay between HSPs, ERK, and AMPK thus forms a signaling network that coordinates mitochondrial function, ROS homeostasis, and cancer cell proliferation.

### 3.3 Regulatory Roles of HSP70 and HSP90 in Bioenergetic Processes

Beyond their well‐established functions in protein folding and stress responses, HSP70 and HSP90 directly participate in cellular bioenergetic processes that sustain tumor growth and survival [97, 98].

#### 3.3.1 HSP70 in Mitochondrial ATP Production and Metabolic Homeostasis

The mitochondrial isoform of HSP70 (mtHSP70/mortalin, encoded by HSPA9) plays an indispensable role in maintaining OXPHOS capacity. By facilitating the import of nuclear‐encoded ETC subunits and TCA cycle enzymes, mtHSP70 ensures the proper assembly and function of complexes I, III, and IV [57, 99]. Depletion of mtHSP70 leads to reduced ATP synthesis, increased ROS production, and activation of AMPK‐dependent metabolic reprogramming [100]. Additionally, cytosolic HSP70 (HSPA1A) has been implicated in maintaining cellular ATP levels under glucose deprivation by stabilizing glycolytic enzymes and facilitating their compartmentalization [101].

#### 3.3.2 HSP90 in Bioenergetic Adaptation

HSP90 exerts bioenergetic control through multiple mechanisms. First, HSP90 stabilizes HIF‐1α, a master transcriptional regulator that orchestrates the switch from OXPHOS to glycolysis under hypoxic conditions [53, 102, 103]. By preventing HIF‐1α degradation, HSP90 promotes the expression of glucose transporters and glycolytic enzymes, ensuring continued ATP production when oxygen is limiting. Second, HSP90 directly interacts with and stabilizes HK2 on the outer mitochondrial membrane, coupling glycolysis with mitochondrial membrane potential and preventing apoptosis [9, 104, 105]. Third, the mitochondrial HSP90 isoform TRAP1 modulates oxidative metabolism by regulating F‐ATP synthase activity and by influencing respiratory‐chain components, including succinate dehydrogenase/complex II, thereby shaping ETC output and mitochondrial ROS production [106]. In glioma models and multiple cancer cell systems, pharmacological targeting of mitochondrial HSP90s disrupts ATP production and activates AMPK‐linked stress signaling, highlighting a central role for HSP90 chaperones in tumor bioenergetic homeostasis [107, 108, 109].

HSP70 and HSP90 cooperate through the HOP/STIP1 co‐chaperone, which physically bridges the two chaperone systems and coordinates sequential client handover from HSP70‐bound holding states to HSP90‐dependent folding/maturation [110, 111]. Given that many HSP90 clients include signaling and metabolic regulators, this HSP70–HSP90 axis may indirectly support bioenergetic adaptation [112, 113]. Moreover, stress‐induced HSF1 activation transcriptionally reinforces the heat‐shock chaperone network, including HSP70 and HSP90, thereby supporting tumor‐cell proteostasis and adaptive survival [114, 115]. Consistently, combined targeting of HSP70 and HSP90 has produced synergistic antitumor effects in preclinical models, suggesting that dual disruption of this chaperone axis may compromise tumor bioenergetic and proteostatic homeostasis.

### 3.4 Regulation of Lipid Metabolism by Heat Shock Proteins

**FIGURE 3:**
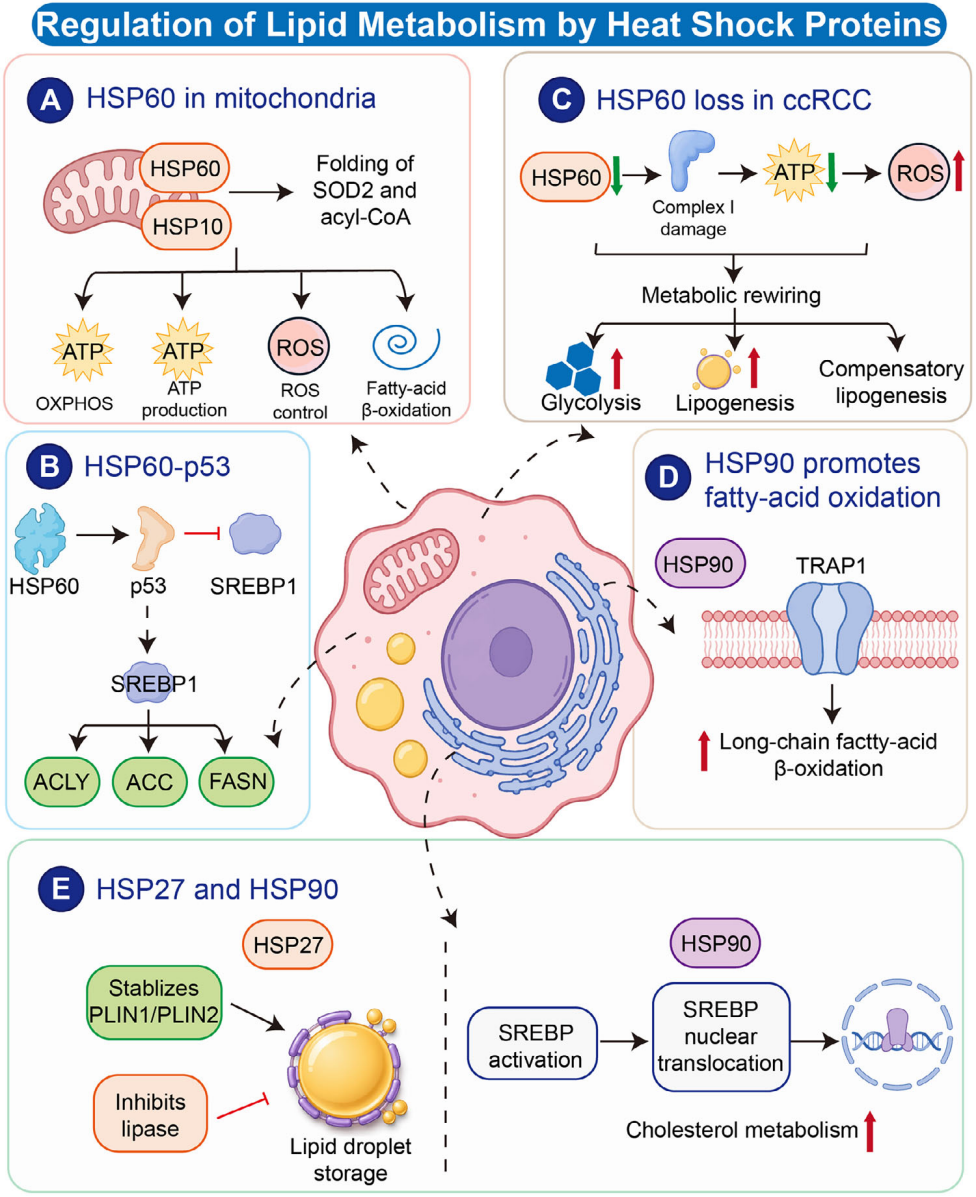
Regulatory roles of HSPs in lipid metabolic reprogramming of cancer cells. (A) The mitochondrial HSP60–HSP10 chaperonin system supports the folding of proteins involved in respiratory activity, ROS control, and fatty‐acid β‐oxidation, thereby maintaining ATP production and mitochondrial lipid catabolism. (B) HSP60 modulates the p53–SREBP1 axis; attenuation of p53‐dependent repression permits SREBP1‐mediated expression of ACLY, ACC, and FASN and promotes fatty‐acid synthesis. (C) Loss of HSP60 in clear cell renal cell carcinoma impairs respiratory‐complex integrity, decreases ATP production, and increases ROS, thereby inducing compensatory glycolysis and lipogenesis. (D) HSP90/TRAP1 maintains mitochondrial proteostasis and metabolic flexibility to regulate oxidative phosphorylation and fatty‐acid β‐oxidation. (E) HSP27 promotes lipid‐droplet storage by stabilizing PLIN1 and PLIN2 and limiting lipase activity, whereas HSP90 promotes SREBP activation and nuclear translocation to support cholesterol metabolism. Regular solid arrows indicate direct molecular events or directional processes; dashed arrows indicate indirect or context‐dependent relationships; blunt‐ended lines indicate inhibition; and bold colored arrows indicate the net increase or decrease in metabolic output. ACC, acetyl‐CoA carboxylase; ACLY, ATP‐citrate lyase; ATP, adenosine triphosphate; ccRCC, clear cell renal cell carcinoma; CPT1, carnitine palmitoyltransferase 1; FASN, fatty‐acid synthase; HSP, heat shock protein; OXPHOS, oxidative phosphorylation; PLIN, perilipin; ROS, reactive oxygen species; SOD2, superoxide dismutase 2; SREBP1, sterol regulatory element‐binding protein 1.

HSPs intricately regulate lipid metabolism, impacting fatty acid synthesis, oxidation, storage, and cholesterol homeostasis (Figure 3).

#### 3.4.1 HSP60 and Mitochondrial Control of Lipid Metabolism

HSP60, in conjunction with its co‐chaperone HSP10, facilitates the proper folding of over 300 mitochondrial matrix proteins, which is critical for mitochondrial function [116]. The structured chaperonin cage of HSP60 is directly responsible for folding key enzymes involved in lipid metabolism, including the antioxidant enzyme SOD2 and acyl‐CoA dehydrogenases [117], both pivotal in oxidative stress responses and fatty acid oxidation.

An apparent paradox has been observed in ccRCC: HSP60 silencing promotes a Warburg‐like phenotype and redirects mitochondrial metabolism from ATP production toward biosynthesis, including enhanced glutamine‐driven de novo lipid synthesis [71]. This metabolic shift is consistent with the broader lipogenic phenotype of ccRCC, in which key fatty‐acid synthesis enzymes such as ACC and FASN are frequently upregulated [118]. This paradox can be explained as an indirect compensatory response. In ccRCC cells, HSP60 silencing disrupts mitochondrial proteostasis and respiratory complex I integrity, resulting in mitochondrial dysfunction, reduced ATP‐producing capacity, and increased ROS generation; these mitochondrial defects then drive adaptive metabolic rewiring toward glycolysis and biosynthesis [71, 119]. In response to mitochondrial energetic and oxidative stress, cancer cells activate adaptive stress‐response pathways, including AMPK‐ and NRF2‐associated programs [120, 121]. However, AMPK activation generally suppresses de novo lipogenesis through ACC phosphorylation and inhibition of SREBP1‐dependent lipogenic transcription. Therefore, the increased ACC and FASN expression observed after HSP60 knockdown is more likely an indirect compensatory metabolic adaptation—potentially involving SREBP1/AKT‐mTOR/HIF‐linked lipogenic programs—rather than a direct consequence of HSP60 chaperoning these enzymes [71, 122].

Beyond compensatory stress responses, HSP60 may influence lipid metabolism through its impact on the p53–SREBP1 lipogenic axis [123]. Wild‐type p53 has been reported to repress SREBP‐1c transcription and thereby limit SREBP1‐dependent lipogenic programs, including ACLY, ACC, and FASN. Therefore, HSP60‐mediated modulation of p53 activity could indirectly release SREBP1‐driven fatty‐acid synthesis, linking mitochondrial chaperone dysfunction to lipogenic rewiring [124]. HSP60 can form complexes with p53 and restrain p53‐dependent transcriptional activity. Because wild‐type p53 has been reported to transcriptionally repress SREBP‐1, whereas SREBP‐1 drives lipogenic genes including ACLY, ACC and FASN, HSP60‐mediated attenuation of p53 may indirectly favor SREBP‐1‐dependent lipogenesis [125, 126].

HSPs also participate in the regulation of fatty acid β‐oxidation by preserving mitochondrial proteostasis and maintaining the stability of enzymes involved in oxidative metabolism. The mitochondrial HSP90 family member TRAP1 acts as a key regulator of metabolic plasticity by coordinating oxidative phosphorylation, reactive oxygen species homeostasis, and substrate utilization, thereby indirectly influencing fatty acid oxidation [127].

#### 3.4.2 HSPs Govern Lipid Storage and Droplet Formation

HSP27 inhibits lipase activity and stabilizes lipid droplet‐coating proteins (PLIN1/PLIN2) [128], promoting intracellular lipid storage. The oligomeric structure and the α‐crystallin domain of HSP27 are crucial for these interactions. For instance, in pancreatic cancer, HSP27 overexpression drives lipid droplet accumulation [129], providing long‐term energy reserves for tumor cells.

#### 3.4.3 HSPs Interact With Lipid Metabolic Signaling Pathways

AMPK activation suppresses fatty acid synthesis while stimulating fatty acid oxidation. In breast cancer, HSP90 inhibitors diminish AMPK activity, attenuating tumor reliance on fatty acid oxidation [130]. Conversely, HSP70 inhibits mTORC1 activity to promote fatty acid oxidation under stress conditions. Dysregulation of the HSP70‐mTOR axis alters lipid metabolic patterns in gliomas [131].

#### 3.4.4 HSPs Critically Regulate Cholesterol Metabolism

HSP90 modulates lipid metabolism by influencing SREBPs [132], master transcriptional regulators of lipogenic enzymes. In hepatocellular carcinoma, HSP90 promotes SREBP nuclear translocation, augmenting cholesterol metabolism to support membrane and hormone synthesis. Notably, p53 directly binds the SREBP‐1 promoter to repress its expression, thereby impeding lipid metabolic flux [133]. The HSP60‐p53 complex further fine‐tunes these pathways, as detailed above, underscoring the multifaceted role of HSPs in lipid metabolic regulation.

### 3.5 Mechanisms of Heat Shock Proteins in Regulating Biosynthetic and Bioenergetic Pathways

HSP27 functions as a mitochondrial‐released protein factor that integrates stress‐sensing signals with cell survival mechanisms. Under conditions of mild oxidative stress, HSP27 is released from mitochondria and subsequently binds to and activates the IκB kinase (IKK) complex in the cytosol [134]. This activation triggers NF‐κB‐dependent expression of survival genes in the nucleus [135], constituting a critical mechanism for host cell survival. In clear cell renal carcinoma (ccRCC), experimental evidence from Teng et al. demonstrated that HSP60 knockdown using specific siRNA significantly increased the protein levels of NRF2 and its downstream target NQO1, accompanied by elevated glutathione (GSH) production and reduced ROS accumulation [71]. Mechanistically, HSP60 loss‐of‐function impairs the proper folding of electron transport chain subunits, leading to mitochondrial dysfunction and increased ROS leakage from complexes I and III. This ROS burst activates the KEAP1‐NRF2 pathway by promoting NRF2 dissociation from its negative regulator KEAP1, allowing NRF2 nuclear translocation and transcriptional activation of antioxidant response elements. The consequent upregulation of GSH biosynthetic enzymes enhances ROS‐scavenging capacity, enabling ccRCC cells to survive under oxidative stress while maintaining rapid proliferation. This compartmentalized regulation—where mitochondrial ETC dysfunction leads to cytosolic/nuclear NRF2 activation—represents a key adaptive mechanism in ccRCC progression.

Furthermore, HSP60 exhibits a close association with glutamine metabolism. Intracellular aspartate levels serve as a rate‐limiting factor for de novo nucleotide synthesis, which is essential for tumor growth [136, 137]. Aspartate can be generated through glucose oxidation, glutamine oxidation, or reductive carboxylation of glutamine [138]. Notably, glutamine oxidation represents the predominant pathway for pyrimidine‐based nucleotide synthesis. In clear cell renal carcinoma, HSP60 silencing activates the MEK/ERK/c‐Myc signaling axis, thereby promoting glutamine‐directed metabolic reprogramming [71]. This metabolic shift drives mitochondria to transition from ATP production to biosynthetic processes, ultimately facilitating tumor progression.

### 3.6 The Dual Role of Reactive Oxygen Species in Metabolic Reprogramming: Compartmentalized Signaling versus Global Oxidative Stress

Reactive oxygen species (ROS) play a paradoxical role in cancer biology, functioning as either signaling molecules that promote proliferation and adaptation or as cytotoxic agents that induce cell death, depending on their concentration, subcellular origin, and duration of exposure [139]. A critical distinction, often overlooked, is between compartmentalized ROS signaling and global oxidative stress. Localized ROS bursts generated at defined subcellular sites can act as second messengers to activate specific redox‐sensitive pathways, whereas widespread ROS accumulation overwhelms antioxidant buffering and causes nonspecific oxidative damage to lipids, proteins, and DNA [140, 141]. HSPs participate in both redox buffering and stress‐response signaling, but how individual HSP family members differentially regulate localized ROS signaling versus global oxidative injury remains incompletely defined [9].

Mitochondria are a major intracellular source of ROS, generated predominantly by electron leakage from the ETC during OXPHOS, particularly at complexes I and III [142]. Under physiological or mildly stressed conditions, low‐level mitochondrial ROS act as localized signaling molecules that activate adaptive pathways, including KEAP1–NRF2 antioxidant signaling, AMPK‐linked metabolic adaptation, and HIF‐1α stabilization [143, 144]. HSPs are positioned to modulate mitochondrial redox signaling. HSP60 maintains mitochondrial proteostasis and respiratory‐chain integrity; in ccRCC models, HSP60 knockdown disrupts respiratory Complex I, increases mitochondrial ROS production, and activates AMPK‐linked adaptive signaling [71, 145]. These data support a model in which HSP60 constrains ETC‐derived ROS and prevents excessive redox stress, although whether this represents compartmentalized signaling rather than global oxidative stress remains to be directly established. Similarly, mtHSP70/mortalin is a core ATPase of the mitochondrial protein‐import machinery and supports mitochondrial protein quality control; its depletion would be expected to impair mitochondrial proteostasis and redox homeostasis, but direct phenocopying of HSP60 loss in ccRCC requires specific experimental validation [57, 146].

When ROS production exceeds antioxidant buffering capacity—because of severe mitochondrial dysfunction, exogenous pro‐oxidants, or impaired chaperone protection—global oxidative stress occurs, leading to lipid peroxidation, protein oxidation/carbonylation, DNA damage, and activation of regulated cell‐death pathways such as apoptosis, ferroptosis, and necroptosis [147, 148]. HSPs help prevent this transition by maintaining proteostasis and supporting antioxidant‐defense networks. HSP27, as an ATP‐independent small HSP with holdase activity, protects cells from oxidative injury and has been linked to preservation of glutathione‐dependent antioxidant capacity; however, direct stabilization of specific enzymes such as GST or catalase should be stated only if supported by the cited primary study [149, 150]. Additionally, HSP27 modulates the IKK/NF‐κB axis in a context‐dependent manner and has been linked to NF‐κB‐dependent induction of antioxidant enzymes such as MnSOD/SOD2 [151]. HSP70 also contributes to redox protection, not by acting as a classical ROS scavenger, but by redox‐sensitive cysteine modifications that tune its chaperone activity and by promoting NRF2/HO‐1‐associated antioxidant responses [152, 153]. When these HSP‐mediated protective mechanisms are insufficient—because of genetic depletion, pharmacological inhibition, or overwhelming oxidative stress—ROS may exceed antioxidant buffering capacity and trigger cell death [154]. This context‐dependent duality may explain why mild or partial interference with HSP networks can elicit adaptive stress signaling, whereas profound loss of chaperone function is cytotoxic.

Beyond their canonical chaperone functions, several HSPs indirectly regulate ROS homeostasis by buffering oxidized or misfolded proteins and supporting antioxidant proteostasis [155]. HSP27/HSPB1 contains a redox‐sensitive cysteine residue whose oxidation can alter its conformation, oligomeric state, and cytoprotective activity. Under mild oxidative or proteotoxic stress, HSP27 functions as an ATP‐independent holdase, forming dynamic oligomers that bind partially unfolded or oxidized proteins and help maintain their solubility [156, 157]. In addition, HSP27 has been linked to ubiquitin–proteasome‐dependent protein clearance and, more recently, to p62/SQSTM1‐associated selective autophagy, suggesting that it coordinates multiple protein quality‐control routes under redox stress [158]. HSP70 contributes to redox homeostasis by supporting glutathione‐dependent antioxidant capacity; HSP70 overexpression has been shown to enhance GPx and glutathione reductase activities under ischemic or hypoxic stress [152, 159]. However, direct stabilization of the GPx or thioredoxin systems by HSP70 remains insufficiently established. HSP90 may indirectly influence antioxidant adaptation through stabilization of HIF‐1α, which cooperates with hypoxia‐ and oxidative‐stress pathways, including NRF2‐associated responses, to regulate mitochondrial and antioxidant homeostasis [160]. Importantly, HSP‐dependent redox control is likely compartmentalized rather than simply ROS‐scavenging. Mitochondrial HSP60 and mtHSP70 maintain mitochondrial proteostasis and respiratory‐chain integrity, thereby limiting excessive ETC‐derived ROS, whereas cytosolic HSP27 and HSP70 buffer oxidatively damaged proteins and support cytosolic redox‐proteostasis networks [161, 162, 163]. This compartmentalized chaperone activity may help preserve localized ROS signaling while preventing diffuse oxidative injury.

The dual role of ROS in tumor promotion versus tumor suppression has important implications for HSP‐targeted therapy. Partial or transient disruption of mitochondrial chaperone function may increase ROS to a level that activates adaptive programs, including AMPK‐ and NRF2‐associated stress responses, thereby potentially supporting metabolic adaptation and therapy resistance [164]. By contrast, sustained or more profound inhibition of HSP60/HSP70, particularly when combined with ROS‐inducing therapies such as doxorubicin, paclitaxel, or radiotherapy, may push ROS beyond the antioxidant buffering capacity of cancer cells and trigger oxidative cell death, including apoptosis and ferroptosis [165]. Thus, the therapeutic efficacy of HSP inhibitors may depend less on simple target engagement and more on whether ROS elevation crosses a cytotoxic threshold rather than remaining within an adaptive signaling range [166]. Future studies should incorporate compartment‐resolved ROS and redox probes—such as MitoSOX or genetically encoded mitochondrial ROS sensors for mitochondrial superoxide/oxidant dynamics, and roGFP/Grx1‐roGFP or roGFP‐Orp1 systems for cytosolic or organelle‐specific redox state—to monitor how HSP inhibition reshapes redox signaling and to guide rational combinations with pro‐oxidant therapies [167, 168].

### 3.7 Substrate Specificity and Integrative Roles of HSPs in Metabolic Reprogramming

A key question is how HSP90, HSP70, and HSP60 achieve substrate specificity in regulating metabolic enzyme stability and function. While all are ATP‐dependent chaperones, their distinct structures and co‐chaperone networks dictate client selection (Table 2). HSP90 preferentially interacts with metastable, signal‐transducing proteins, including specific metabolic enzymes such as HK2 and PKM2, stabilizing their activated states. HSP70, with its broad substrate‐binding domain, engages hydrophobic segments of unfolded polypeptides; its specificity is often directed by HSP40 co‐chaperones, which deliver specific clients such as glucose transporters. HSP60 functions as a folding cage for a select subset of proteins, primarily essential mitochondrial matrix enzymes and ETC complex subunits.

**TABLE 2:** HSP‐regulated metabolic pathways, mechanisms, and representative translational evidence.

Metabolic pathway/process	HSP regulator(s)	Direct target(s) or client process	Mechanistic link	Representative translational evidence	Selected refs.
Aerobic glycolysis/Warburg effect	HSP90α/β; HSP70; HSP60	HK2, PKM2, GLUT1, HIF‐1α; p53/TIGAR axis	HSP90 stabilizes HK2 and HIF‐1α and regulates glycolytic client complexes, whereas HSP60 modulates the p53–TIGAR axis.	Human tumor datasets and cancer‐cell studies link HSP90/HSP70 expression to glycolytic enzymes and poor prognosis; HSP60‐p53 targeting is supported by prostate cancer EV‐siRNA data	[12, 13, 53, 83, 123]
Mitochondrial protein import and OXPHOS	HSP60/HSP10; mtHSP70/mortalin; TRAP1	ETC complexes I/III/IV; mitochondrial matrix proteins; F‐ATP synthase/channel regulation	HSP60 folds mitochondrial matrix and ETC components; mtHSP70 drives import of nuclear‐encoded mitochondrial proteins; TRAP1 tunes OXPHOS and mitochondrial stress signaling	Pancreatic cancer and glioma models support HSP60/TRAP1‐dependent mitochondrial bioenergetics; mitochondrial‐targeted HSP90 inhibition shows antitumor activity in glioma models	[57, 86, 88, 106, 107]
Lipid metabolism and fatty‐acid oxidation	HSP60/HSP10	SIRT3‐associated FAO regulation, acyl‐CoA dehydrogenases, mitochondrial proteostasis and cholesterol metabolism.	Chaperonins support folding of FAO enzymes; small HSP holdase activity stabilizes lipid‐droplet‐associated proteins; ER HSP70 coordinates UPR‐driven metabolic adaptation	Disease and tumor‐context data connect HSP60/HSP27/GRP78 to lipid storage, mitochondrial fatty‐acid metabolism and therapy resistance, but cancer‐specific causality remains incompletely resolved	[93, 117, 127, 145]
Redox buffering, ferroptosis and stress survival	HSP27/HSPB1; HSP60; HSP70	ROS‐buffering networks; NF‐κB; cGAS‐STING; ferroptosis‐related programs	sHSP oligomers buffer oxidative proteotoxicity; HSP27 modulates NF‐κB/ferroptosis sensitivity; HSP60 posttranslational modification and oligomerization influence inflammatory and immune‐metabolic signaling	Recent studies implicate HSPB1 in squamous‐cell carcinoma ferroptosis resistance and HSP60 methylation/oligomerization in antitumor immunity	[32, 63, 91, 151, 152]
Proteostasis‐metabolism coupling and client triage	HSP70‐HSP90‐HOP/STIP1 axis; HSP40; HSP110	Metabolic enzymes, kinases, p53 variants and misfolded mitochondrial‐destined proteins	Co‐chaperones determine whether clients are folded, held, transferred to HSP90, imported into mitochondria or degraded	Structural and cellular studies define HSP70‐HSP90 cascade control; therapeutic disruption is attractive but requires improved specificity and biomarkers	[26, 48, 50, 110, 111]
Tumor immunity and immune‐metabolic crosstalk	Extracellular/membrane HSP70; HSP60; GRP78; gp96	DAMP signaling, antigen chaperoning, membrane‐HSP targets, cytokine release and immune‐cell activation	Extracellular HSPs can promote antigen presentation or inflammatory signaling; membrane HSP70/GRP78 provide tumor‐associated targets for antibodies/CAR‐T strategies	HSPPC‐96/vitespen has clinical vaccine experience; GRP78‐ and HSP70‐directed CAR‐T approaches remain largely preclinical; specificity and on‐target/off‐tumor risk require careful evaluation	[4, 236, 237, 238, 240]

Abbreviations: ATP, adenosine triphosphate; CAR‐T, chimeric antigen receptor T‐cell; cGAS, cyclic GMP–AMP synthase; DAMP, damage‐associated molecular pattern; ER, endoplasmic reticulum; ETC, electron transport chain; EV, extracellular vesicle; FAO, fatty‐acid oxidation; F‐ATP synthase, F‐type ATP synthase; GLUT1, glucose transporter 1; gp96, glycoprotein 96; GRP78, 78‐kDa glucose‐regulated protein; HIF‐1α, hypoxia‐inducible factor 1α; HK2, hexokinase 2; HOP, HSP70–HSP90 organizing protein; HSP, heat shock protein; HSPA5, heat shock protein family A member 5; HSPB1, heat shock protein family B member 1; HSPPC‐96, heat shock protein peptide complex 96; mtHSP70, mitochondrial HSP70; NF‐κB, nuclear factor κB; OXPHOS, oxidative phosphorylation; p53, tumor protein p53; PKM2, pyruvate kinase M2; PLIN, perilipin; ROS, reactive oxygen species; sHSP, small heat shock protein; siRNA, small interfering RNA; SIRT3, sirtuin 3; STING, stimulator of interferon genes; STIP1, stress‐induced phosphoprotein 1; TIGAR, TP53‐induced glycolysis and apoptosis regulator; TRAP1, TNF receptor‐associated protein 1; UPR, unfolded protein response.

These chaperone systems do not operate in isolation. Functional interplay exists, for example, where HSP70 prefolds substrates later transferred to HSP60 for final assembly. This collaborative network allows for integrated control over the cancer proteome, ensuring metabolic plasticity. The coordinated action of HSPs enables tumors to adapt to metabolic demands, balance biosynthetic and energetic outputs, and withstand microenvironmental stress, positioning the chaperone network as a critical therapeutic target.

To clarify the mechanistic logic of this review, we propose a three‐level framework linking HSP structure, metabolic function, and cancer phenotype. Structurally, different HSP families possess distinct chaperone modules—such as the ATP‐dependent client‐maturation cycle of HSP90, the NBD–SBD allosteric cycle of HSP70, the mitochondrial folding chamber of HSP60, and the ATP‐independent holdase activity of HSP27/HSPB1 [104, 108]. Functionally, these properties support key metabolic processes, including glycolytic enzyme stability and flux, mitochondrial protein import, ETC integrity, OXPHOS maintenance, ROS buffering, and stress‐associated lipid storage [169, 170]. Phenotypically, these metabolic effects converge to promote tumor‐cell proliferation, survival under oxidative or proteotoxic stress, and metastatic adaptation. Thus, linking structural chaperone mechanisms to metabolic outputs and cancer phenotypes provides a concise framework for understanding how HSPs coordinate metabolic reprogramming and for developing rational HSP‐targeted therapeutic strategies [171].

### 3.8 Advances in Heat Shock Protein‐Targeted Therapeutics: Current Research Landscape

The therapeutic targeting of HSPs seeks to exploit the dependence of cancer cells on chaperone‐supported proteostasis while avoiding unacceptable disruption of protein homeostasis in normal tissues. This section critically evaluates pharmacological and immunological strategies directed against HSP90, HSP70, HSP60, and their associated client and co‐chaperone networks. The discussion first considers the major mechanistic classes of HSP‐targeted agents and their translational status, followed by family‐specific inhibitors, isoform‐selective compounds, and approaches that disrupt chaperone–client or chaperone–co‐chaperone interfaces. It then examines HSP‐directed CAR‐T cells, monoclonal antibodies, cancer vaccines, and rational combinations with metabolic or immune therapies. For each strategy, mechanistic and preclinical evidence is considered alongside clinical phase, antitumor activity, toxicity, adaptive heat‐shock responses, resistance mechanisms, and biomarker requirements, thereby clarifying the factors that have limited broad clinical implementation and the features required to improve therapeutic selectivity and patient stratification.

### 3.9 Overview of Heat Shock Protein‐Targeted Pharmacological Agents

In the field of oncology, HSPs have emerged as compelling therapeutic targets due to their multifaceted roles in modulating hallmark characteristics of cancer. These include maintaining proliferative signaling, evading growth suppressors, resisting cell death, inducing angiogenesis, activating invasion and metastasis, reprogramming energy metabolism, and avoiding immune destruction [9]. Among the HSP family, HSP90, HSP70, and HSP60 represent the most prominent members with distinct molecular functions. HSP60 primarily provides a sequestered environment for initial protein folding; HSP70 orchestrates protein unfolding, degradation, and refolding processes, while HSP90 facilitates the recognition and maturation of partially folded proteins during late‐stage folding or targets them for proteasomal degradation [124].

Currently, HSP90 stands as the only validated HSP target that has successfully transitioned into clinical applications for cancer treatment, with several HSP90 inhibitors having received regulatory approval. Although HSP70 has demonstrated promising potential in preclinical studies and generated considerable patent activity, its therapeutic development faces substantial translational challenges. Comparatively, while some progress has been made in HSP60‐targeted drug discovery, research enthusiasm and clinical advancement in this area remain significantly overshadowed by the HSP90 paradigm (Table 3). The disparate clinical progress among these HSP families stems from fundamental differences in their druggability, the specificity of their client proteins, and the nature of the challenges encountered during drug development.

**TABLE 3:** Clinical and preclinical HSP‐targeted agents in cancer: molecular mechanism, translational status, efficacy, toxicity, and major limitations.

Agent/strategy	Molecular target and binding modality	Mechanism of action	Tumor type	Clinical phase/current status	Trial identifier/principal evidence	Key efficacy or translational finding	Major toxicity and translational limitations	Refs.
Pimitespib (TAS‐116; Jeselhy)	HSP90α/β N‐terminal ATP‐binding pocket; orally bioavailable inhibitor	Blocks the ATP‐dependent HSP90 cycle and promotes degradation of oncogenic client proteins; combination with nivolumab may reduce regulatory T‐cell activity.	Advanced GIST; CRC and other solid tumors	Phase III completed; approved in Japan for previously treated GIST. NCT05245968 phase I combination study: active, not recruiting.	CHAPTER‐GIST‐301; EPOC1704/UMIN000032801; NCT05245968	In phase III GIST, median PFS was 2.8 versus 1.4 months with placebo (HR 0.51). TAS‐116 plus nivolumab produced a 16% ORR in MSS CRC without prior checkpoint inhibition.	Diarrhea occurred in 74.1% and grade ≥3 diarrhea in 13.8% in CHAPTER‐GIST‐301. Approval remains geographically and indication restricted; resistance and biomarker‐based selection remain unresolved.	[189, 190, 222]
SNX‐5422 (PF‐04929113)	Pan‐HSP90 N‐terminal ATP‐binding pocket; oral prodrug of SNX‐2112	Depletes HSP90‐dependent oncogenic clients and induces HSP70 as a pharmacodynamic marker of target engagement.	Advanced solid tumors, lymphomas, and hematologic malignancies	Phase I completed; broader clinical development discontinued or terminated; no approval	NCT00644072 and related early‐phase studies	In a solid‐tumor/lymphoma phase I study, no objective responses were observed; 47% achieved stable disease. A separate hematologic phase I study reported one partial response and prolonged stabilization in one patient.	Grade 3 diarrhea, AST elevation, thrombocytopenia, and nonseptic arthritis were observed. Development was limited by ocular/retinal toxicity concerns and modest efficacy.	[205, 206]
Tanespimycin (17‐AAG)	Pan‐HSP90 N‐terminal ATP‐binding pocket; geldanamycin derivative	Destabilizes HER2 and other HSP90 clients, promoting ubiquitin‐proteasome‐dependent degradation.	Trastuzumab‐refractory HER2‐positive metastatic breast cancer; other advanced cancers	Phase II completed; not approved	Modi et al. phase II trial of tanespimycin plus trastuzumab	Combination therapy achieved an ORR of 22%, clinical benefit rate of 59%, median PFS of 6 months, and median OS of 17 months.	Hepatotoxicity, formulation and pharmacokinetic constraints, and compensatory heat‐shock responses limited broad development; activity was concentrated in a selected client‐dependent tumor context.	[172, 174, 178]
Alvespimycin (17‐DMAG)	Pan‐HSP90 N‐terminal ATP‐binding pocket; water‐soluble geldanamycin derivative	Induces degradation of HSP90 clients and was evaluated with trastuzumab to intensify HER2 depletion	HER2‐positive metastatic breast cancer and other advanced solid tumors	Phase I completed; not approved	Jhaveri et al. phase I dose‐escalation trial	One partial response and seven cases of stable disease lasting 4–10 months were observed, all in HER2‐positive metastatic breast cancer	Dose‐limiting grade 3 congestive heart failure and reversible grade 3 keratitis occurred; other grade 3 toxicities included fatigue, diarrhea, myalgia, and back pain	[207]
PET‐16	HSP70 substrate‐binding‐domain allosteric pocket; preferential binding to the non‐ATP‐bound state	Disrupts HSP70 allosteric cycling and client interactions, reducing phospho‐FAK and mutant BRAF and impairing invasion and metastatic signaling	Melanoma	Preclinical; no oncology NCT identified	Cellular, xenograft, and metastasis models	Reduced melanoma invasion and lung metastasis and enhanced the durability of BRAF‐inhibitor responses in experimental models	Human pharmacokinetics, therapeutic window, and clinical safety are unknown; the compound has potency and chemical‐development liabilities	[175]

Abbreviations: ATP, adenosine triphosphate; CLL, chronic lymphocytic leukemia; CRC, colorectal cancer; GIST, gastrointestinal stromal tumor; HER2, human epidermal growth factor receptor 2; HSP, heat shock protein; MSS, microsatellite‐stable; NCT, ClinicalTrials.gov identifier; NSCLC, non–small cell lung cancer; NTD, N‐terminal domain; TP53, tumor protein p53.

Despite decades of research, the translational development of HSP inhibitors has produced both encouraging signals and important setbacks. Preclinical models have been essential for validating HSP‐dependent oncogenic clients and resistance mechanisms, but clinical efficacy has often been limited by toxicity and adaptive heat‐shock responses. For example, the HSP90 inhibitor 17‐AAG/tanespimycin showed strong rationale in HER2‐driven breast cancer by promoting ErbB2/HER2 degradation and later demonstrated clinical activity in combination with trastuzumab in HER2‐positive metastatic breast cancer [172]. In BCR‐ABL‐driven leukemia, HSP90 inhibition prolonged survival in BCR‐ABL‐T315I mouse models and suppressed leukemic stem cells [173]. However, early clinical studies of 17‐AAG revealed dose‐limiting toxicities, including liver toxicity, and N‐terminal HSP90 inhibition can induce HSP70/HSP27 expression through the heat‐shock response, potentially protecting residual tumor cells [174]. Similarly, HSP70 inhibition with PET‐16 reduced phospho‐FAK, impaired melanoma invasion, and suppressed lung metastasis in B16‐F10 models, supporting the therapeutic potential of targeting HSP‐dependent metastatic signaling [175]. These examples underscore the critical need for better predictive animal models and the incorporation of pharmacodynamic biomarkers in early‐phase clinical trials.

### 3.10 Heat Shock Protein 90 (HSP90) Inhibitors

As one of the most extensively studied members of the heat shock protein family, HSP90 plays a pivotal role in stabilizing numerous client proteins. Many of these clients are key drivers of tumorigenesis and metabolic reprogramming, including kinases, transcription factors, and enzymes like HK2 and PKM2. Notably, targeting HSP90 disrupts multiple biological pathways involved in tumorigenesis, offering broader therapeutic implications compared with conventional single‐target approaches [176]. HSP90 inhibitors can induce degradation of most oncoproteins, thereby reducing the likelihood of tumor drug resistance [177]. These advantages have established HSP90 as a prominent target in anticancer research.

The first HSP90 ATPase inhibitor, the natural product geldanamycin, was discovered in 1997 and entered research investigations. The mechanism of HSP90 ATPase inhibitors involves ATP mimicry, where they competitively bind to the ATP‐binding pocket in the N‐terminal domain of HSP90, thereby blocking its ATP‐dependent conformational changes. This process interferes with the normal chaperone cycle, preventing proper folding of overexpressed immature oncoproteins, which are subsequently recognized and degraded by the ubiquitin‐proteasome system (UPS) [178]. The first‐generation HSP90 ATPase inhibitors include geldanamycin and its derivatives, such as 17‐AAG, 17‐DMAG, IP‐504, and IP‐493. However, development of these compounds was ultimately discontinued due to their inherent hepatotoxicity [179]. The second class comprises resorcinol‐based inhibitors, whose core resorcinol scaffold forms hydrogen bonds with Asp93 and Thr184 in the N‐terminal ATP‐binding pocket of HSP90, demonstrating high target affinity [180]. While these small molecules exhibit lower toxicity and better tolerability, their therapeutic efficacy remains suboptimal. The third class consists of purine‐based compounds, which offer advantages such as high oral bioavailability, selective retention in tumor tissues, and blood–brain barrier permeability [181]. Nevertheless, clinical trials failed to demonstrate significant therapeutic benefits, leading to discontinuation after phase I/II studies. The fourth class includes benzamide derivatives such as SNX‐5422, SNX‐2112, TAS‐116, and XL‐888 [182]. Although SNX‐5422 demonstrated antiproliferative activity in over 20 solid and hematological tumors and induced degradation of various protumor molecules, its phase II clinical trials were halted due to dose‐limiting ocular toxicity and potential irreversible retinal damage [183]. A critical and persistent challenge for N‐terminal ATPase inhibitors has been the nonselective degradation of client proteins and the compensatory upregulation of HSP70 expression, which triggers a heat shock response that diminishes drug efficacy. This compensatory induction of HSP70, a pro‐survival chaperone, represents a major adaptive resistance mechanism that has plagued the clinical efficacy of many HSP90 inhibitors. Consequently, small‐molecule ATPase inhibitors have faced persistent challenges in clinical translation.

In mammalian cells, the HSP90 family comprises four isoforms: HSP90α/β, GRP94, and TRAP1 [184]. To achieve precise tumor cell targeting, researchers have focused on developing isoform‐selective HSP90 inhibitors. However, single‐isoform inhibition has proven insufficient to meet clinical demands, and the limited foundation in druggability research has hindered the identification of clear clinical indications. Thus, strategies targeting individual isoforms remain inadequate to replace broad‐spectrum HSP90 inhibitors.

With advancing understanding of chaperone system mechanisms, increasing attention has been directed toward the critical regulatory roles of cochaperones, including substrate recognition and presentation, conformational stabilization, and enzymatic catalysis. Disrupting protein–protein interactions (PPIs) between HSP90 and its cochaperones to precisely modulate the chaperone cycle has emerged as a novel drug design strategy. Inhibitors targeting HSP90‐cochaperone interactions, such as those with CDC37 and AHA1, represent a promising avenue to achieve greater selectivity and potentially circumvent the compensatory heat shock response associated with ATP‐competitive inhibitors. Several studies have explored PPI inhibitors targeting three key cochaperones: CDC37, AHA1, and HOP. Targeting these specific PPIs represents a promising strategy to overcome the limitations of pan‐HSP90 inhibition. CDC37, a kinase‐specific cochaperone, facilitates the folding and maturation of client kinases by recognizing and recruiting unfolded kinase client proteins to HSP90 [171]. Research indicates that CDC37 is overexpressed in various cancers [185], making the HSP90‐CDC37 PPI an attractive therapeutic target. For instance, Celastrol exerts antiproliferative effects by binding to the NTD region of CDC37 and disrupting HSP90‐CDC37 interactions [186]. AHA1, another critical cochaperone, acts during the late stages of the chaperone cycle by enhancing HSP90 ATPase activity and accelerating the formation of its N‐terminal closed conformation [187]. Several small‐molecule inhibitors targeting HSP90‐AHA1 PPI, including SEW84, A12, A16, and TL‐2‐8, have entered biological testing. Additionally, the cochaperone HOP bridges HSP70 and HSP90, transferring nascent polypeptides from the HSP70 complex to HSP90 [188], offering a new research direction for targeting HSP90‐HOP PPI.

Since the first HSP90 inhibitor entered clinical trials in 1999, numerous candidates have progressed through clinical development, with over 10 reaching various trial stages. Notably, pimitespib (TAS‐116) became the first HSP90 inhibitor approved in Japan and China (2022) for treating gastrointestinal stromal tumors [189, 190], marking a significant milestone for the field. Although market penetration remains limited, the robust pipeline of HSP90‐targeted agents continues to hold promise for future clinical breakthroughs. Furthermore, emerging modalities such as proteolysis‐targeting chimeras (PROTACs) designed to degrade HSP90 itself or its specific oncogenic clients, as well as antibodies targeting extracellular HSP90, are being explored to overcome the limitations of traditional small‐molecule inhibitors and shape the future of the field.

To date, more than 20 HSP90 inhibitors have entered clinical trials (Phase I–III), yet only pimitespib has received regulatory approval, highlighting substantial translational hurdles. A pooled analysis of clinical trials with first‐generation inhibitors revealed objective response rates of less than 10% in unselected patient populations, despite robust target engagement and client protein degradation in surrogate tissues. This disconnect between pharmacodynamic effect and clinical efficacy is attributable to several factors: (i) compensatory upregulation of HSP70 and other pro‐survival chaperones; (ii) dose‐limiting toxicities including hepatotoxicity, ocular toxicity, and gastrointestinal adverse events; (iii) poor physicochemical properties leading to suboptimal tumor penetration; and (iv) lack of validated predictive biomarkers. Emerging evidence from Phase II trials of second‐generation inhibitors suggests that combinatorial approaches—particularly with BRAF inhibitors in melanoma or with immune checkpoint inhibitors in mismatch repair‐proficient colorectal cancer—may overcome resistance [175, 190]. These findings underscore the need for rational combination strategies and biomarker‐driven patient selection rather than continued pursuit of HSP90 monotherapy in unselected populations.

### 3.11 Heat Shock Protein 60 (HSP60) Inhibitors

Current research on small‐molecule HSP60 inhibitors has primarily focused on several compounds including BF844, Phenoxyacetanilide, KHS‐101, Suvanine, Epolactaene, and Myrtucommulone. In contrast to HSP90, the development of clinically viable HSP60 inhibitors is still in its infancy, largely due to challenges in identifying well‐defined, druggable pockets and a comprehensive understanding of its specific oncogenic clientele. These inhibitors exhibit antitumor effects through mechanisms such as inducing metabolic reprogramming or triggering apoptosis in tumor cells.

KHS101 has emerged as a promising therapeutic candidate for glioblastoma multiforme (GBM). Its mechanism of action principally involves disrupting tumor cell energy metabolism. Specifically, KHS101 selectively binds to the mitochondrial chaperonin HSP60, inducing the aggregation of both HSP60 and its client proteins, thereby destabilizing the HSP60‐centered protein network [191]. This interference significantly impacts multiple enzymes critical to energy metabolism, including glycolytic enzymes (aldolase and fructose‐bisphosphate aldolase A) and tricarboxylic acid (TCA) cycle components (particularly dihydrolipoyllysine‐residue succinyltransferase, DLST). Furthermore, it markedly impairs the function of ATP synthase F1 subunit α (ATP5A1), a key enzyme in oxidative phosphorylation [192]. Collectively, these effects lead to selective impairment of both mitochondrial bioenergetics and glycolysis in GBM cells. Preclinical studies using mouse xenograft models have demonstrated both the remarkable efficacy and favorable safety profile of KHS101, underscoring its potential as an anticancer agent worthy of further investigation [191].

HSP60 plays a pivotal role in apoptosis regulation. 6‐Shogaol (6‐SH) has been identified as a potential therapeutic candidate for non–small cell lung cancer (NSCLC) through its HSP60‐inhibitory activity. Mechanistic studies reveal that 6‐SH interacts with critical HSP60 residues (Ile150, Pro33, and Gly53) via hydrophobic interactions and hydrogen bonding, thereby reducing HSP60 protein stability and promoting its degradation through the proteasome‐mediated pathway. Notably, HSP60 has been shown to form complexes with survivin, stabilizing this anti‐apoptotic protein [193]. The therapeutic effects of 6‐SH include disruption of mitochondrial membrane potential, concomitant with downregulation of anti‐apoptotic proteins (Bcl‐2 and survivin) and upregulation of pro‐apoptotic Bax, suggesting mitochondrial‐mediated apoptosis induction [194]. Another HSP60 inhibitor, Myrtucommulone A, has been shown to induce mitochondrial dysfunction and cytochrome c release in HL‐60 leukemia models, supporting HSP60 as a potential apoptosis‐related vulnerability [195, 196]. This apoptotic effect appears related to mitochondrial dysfunction following HSP60 inhibition, manifested by impaired malate dehydrogenase refolding activity and aggregation of lon protease‐like protein and leucine‐rich PPR motif‐containing protein [195].

Although the precise mechanisms of these inhibitors remain incompletely understood and their selectivity requires improvement, they represent promising leads for HSP60‐targeted cancer therapy.

### 3.12 Heat Shock Protein 70 (HSP70) Inhibitors

In contrast to the well‐developed pharmacological landscape of HSP90 inhibitors, the development of HSP70 inhibitors faces substantial challenges. A primary obstacle is the lack of a well‐defined, deep hydrophobic pocket suitable for high‐affinity small‐molecule binding, unlike the more druggable N‐terminal ATP‐binding site of HSP90. Additionally, the high degree of homology and functional redundancy among HSP70 isoforms complicates the achievement of selective inhibition. Although extensive research and comprehensive reviews have identified HSP70 as a promising therapeutic target in oncology, no HSP70 inhibitor with significant pharmacological potential has been clinically validated to date. Currently, small‐molecule compounds primarily serve as chemical probes for investigating the biological mechanisms of HSP70 through targeted inhibition.

The PPIs between HSP70 and its client proteins represent a viable strategy for developing novel anticancer therapies. For instance, the HSP70‐Bim PPI has been demonstrated to suppress apoptosis by modulating the conformational dynamics of oncogenic client proteins AKT and Raf‐1 in cancer cells [197]. A recent study reported S1g‐6, a novel HSP70 inhibitor that selectively binds to the nucleotide‐binding domain (NBD) of HSP70, effectively disrupting the HSP70‐Bim interaction. This inhibition leads to decreased phosphorylation and protein levels of AKT and Raf‐1, ultimately inducing apoptotic cell death. Notably, S1g‐6 exhibited potent pro‐apoptotic effects in chronic myeloid leukemia (CML) cell lines (BV173 and KCL22) while showing minimal cytotoxicity in normal tissue‐derived cell lines (HEK293 and BaF3) [198].

Shin et al. developed Az‐TPP‐O3, a mitochondria‐targeted inhibitor specifically designed to disrupt the Mortalin‐p53 PPI. This compound facilitates mitochondrial internalization, subsequently inducing Bax‐mediated mitochondrial outer membrane permeabilization (MOMP). The resulting cytochrome c (Cyt‐C) release promotes apoptosome assembly and caspase cascade activation, culminating in programmed cell death [199]. Similarly, solasonine, a steroidal alkaloid glycoside derived from Solanum xanthocarpum, has been identified as another Mortalin‐p53 PPI inhibitor that induces apoptosis in human hepatocellular carcinoma cell lines (HepG2 and Hep3b) through analogous mechanisms [200].

The elevated expression of HSP70 in melanoma strongly correlates with tumor aggressiveness. Murphy and colleagues investigated the therapeutic potential of PET‐16, an HSP70 inhibitor, in melanoma treatment. Their study revealed that pFAK, a novel HSP70 client protein, undergoes degradation upon PET‐16‐mediated HSP70 inhibition, consequently impairing melanoma metastatic capacity. In vivo murine models further validated the antimigration, anti‐invasion, and antimetastasis effects of PET‐16 [175]. In a separate investigation, epoxyquinol was characterized as a novel HSP70 modulator that suppresses its ATPase activity through direct binding. Given the established association between plasma membrane‐localized HSP70 and tumor metastasis, epoxyquinol represents a promising anti‐metastatic agent with potential clinical applications [201, 202]. Furthermore, GRP78 (glucose‐regulated protein 78, also known as HSPA5), an endoplasmic reticulum (ER)‐resident member of the HSP70 family, plays pleiotropic roles in tumor progression. As a master regulator of the URP, GRP78 is frequently overexpressed in various cancers, where it promotes cell survival under ER stress conditions commonly encountered in rapidly proliferating tumors due to hypoxia, nutrient deprivation, and acidic pH [203]. The indole‐based methanone derivative IKM5 has been shown to significantly downregulate GRP78 expression in various breast cancer cell lines, thereby attenuating their invasive potential. Mechanistic studies indicate that IKM5 directly binds to GRP78, reducing its expression and disrupting its interaction with TIMP‐1, a negative regulator of matrix metalloproteinases (MMPs). Preliminary evaluations of IKM5 demonstrate favorable safety profiles and pharmacokinetic properties, warranting further investigation into its potential as an anticancer therapeutic agent [204].

### 3.13 Challenges, Limitations, and Future Perspectives

The mechanistic dependence of cancer cells on HSP‐supported proteostasis provides a strong rationale for therapeutic targeting, biomarker development, and rational combination strategies. However, the transition from mechanistic insight and preclinical activity to reproducible clinical benefit remains incomplete. This section evaluates the principal biological, pharmacological, and clinical barriers limiting HSP‐directed interventions and subsequently discusses future priorities in biomarker validation, selective drug development, structural and computational discovery, combination therapy, HSP‐directed immunotherapy, and molecularly informed patient stratification.

### 3.14 Major Challenges and Translational Limitations

HSP‐targeted strategies have demonstrated considerable activity in preclinical cancer models by destabilizing oncogenic client proteins, disrupting proteostasis, altering metabolic adaptation, and sensitizing tumors to other treatments. Nevertheless, their clinical performance has been heterogeneous. Among HSP90 inhibitors, pimitespib has received regulatory approval in Japan for gastrointestinal stromal tumors refractory to standard treatment; however, this success remains restricted to a specific disease setting and does not constitute broad clinical validation of HSP90 inhibition across malignancies [189, 190]. Many other HSP90 inhibitors have demonstrated target engagement without producing durable responses in unselected patient populations, whereas agents targeting HSP70 and HSP60 remain predominantly at the preclinical or early translational stage. These findings indicate that HSP inhibition is unlikely to function as a broadly effective, histology‐independent strategy and may instead require tumors with defined dependence on particular HSP isoforms, clients, or co‐chaperone complexes.

A major obstacle is the limited therapeutic window associated with inhibiting chaperones that are also required for normal cellular proteostasis. First‐generation geldanamycin derivatives were constrained by hepatotoxicity, formulation limitations, and unfavorable pharmacokinetic properties, while several later compounds encountered ocular toxicity, insufficient exposure, or limited efficacy. Pan‐HSP90 inhibition may also activate heat shock factor 1 and increase the expression of HSP70 and other stress‐responsive chaperones, thereby partially restoring proteostasis and attenuating antitumor activity. These adaptive responses indicate that biochemical target engagement does not necessarily predict durable clinical benefit and support the development of isoform‐selective, allosteric, compartment‐specific, or client‐selective strategies.

A second major challenge is the biological heterogeneity of HSP dependence [205]. HSP functions vary according to tumor lineage, molecular genotype, metabolic state, subcellular localization, client repertoire, co‐chaperone composition, and microenvironmental stress. The same HSP may therefore promote tumor survival in one cellular context but exert neutral or tumor‐suppressive effects in another. HSP60/HSPD1 illustrates this context dependency particularly clearly, as its reported effects differ among cancer types and may be determined by mitochondrial localization, posttranslational modification, client availability, and the magnitude of oxidative or metabolic stress. Such heterogeneity limits the interpretation of total HSP expression and argues against using a single chaperone marker as a universal criterion for therapeutic selection.

The lack of validated predictive biomarkers represents an additional barrier. Candidate pharmacodynamic indicators—including HSP70 induction, depletion of HSP90 client proteins, and suppression of downstream signaling—can confirm biological activity but have not consistently identified patients who derive durable clinical benefit [206, 207]. Pharmacodynamic target engagement must therefore be distinguished from predictive enrichment. Total HSP expression is unlikely to provide sufficient discriminatory value because it does not reflect isoform activity, subcellular distribution, co‐chaperone composition, client dependence, or the magnitude of adaptive heat‐shock signaling. More informative stratification models may need to integrate tumor‐specific oncogenic clients, genomic alterations, HSP–co‐chaperone complexes, metabolic dependencies, extracellular or membrane‐associated HSP expression, and early treatment‐induced pharmacodynamic changes. Patient stratification is further complicated by the absence of standardized thresholds for defining HSP dependence. A tumor may express high levels of an HSP without being functionally dependent on that chaperone, whereas a tumor with moderate expression may remain highly dependent on a specific unstable client protein. Future clinical trials should therefore combine histological classification with molecular and functional evidence of chaperone dependence. Biomarker‐enriched designs, paired tumor biopsies, serial blood sampling, and longitudinal assessment of client depletion and heat‐shock‐response activation may help distinguish inadequate drug exposure, adaptive compensation, and loss of client dependence as separate causes of treatment failure.

Current evidence also has important methodological limitations. Many mechanistic studies rely on established cell lines, short‐term pharmacological exposure, or conventional xenografts that do not fully reproduce intratumoral heterogeneity, immune–metabolic interactions, drug distribution, or adaptive resistance in patients. Moreover, metabolic changes following HSP inhibition may result either from direct destabilization of a metabolic client or from secondary consequences of global proteotoxic stress. Future studies should therefore combine genetic perturbation, selective pharmacological tools, rescue experiments, temporal metabolic profiling, patient‐derived organoids, immunocompetent models, and longitudinal clinical specimens. Such approaches will be essential for distinguishing direct and therapeutically actionable HSP dependencies from nonspecific stress responses and for developing clinically informative patient‐selection strategies.

### 3.15 Future Research Directions and Innovative Perspectives

Future research should prioritize the clinical validation of HSP‐based biomarkers, the development of selective therapeutic modalities, rational combination strategies, HSP‐directed immunotherapies, and biomarker‐guided patient stratification. Progress in these areas will require clearer distinction between tumor‐specific HSP dependencies and broader stress responses that also occur in normal tissues.

### 3.16 Advanced Biomarker Discovery: Integrating Liquid Biopsy and Circulating HSPs

HSP expression patterns are associated with tumor type, disease stage, and clinical outcome [9]. Elevated HSP90 expression has been linked to aggressive phenotypes and poor prognosis in breast, lung, colorectal, and other cancers [208]. Liquid‐biopsy approaches may extend these tissue‐based observations by detecting HSPs in serum, circulating tumor cells, or extracellular vesicles [209]. Circulating HSP70 levels have been associated with tumor burden, metastatic dissemination, and adverse outcomes in several solid tumors [210], whereas circulating HSP90α has been investigated as a complementary diagnostic marker for hepatocellular carcinoma [211]. However, circulating HSPs are not cancer‐specific and may also increase during inflammation, autoimmune disease, or tissue injury. Their measured concentrations are additionally influenced by preanalytical conditions, assay platforms, and whether the detected protein is free, complexed, membrane‐associated, or extracellular‐vesicle‐associated [212]. Tumor‐derived exosomes may provide more stable and biologically informative HSP signals because their molecular cargo reflects features of the parental tumor cells [213]. HSP70 and HSP90 are frequently present in tumor‐derived exosomes and participate in intercellular communication, immune regulation, and therapeutic resistance [214, 215]. Vesicular encapsulation protects HSP cargo from extracellular degradation, and exosomal surface markers may permit enrichment of tumor‐derived vesicle populations [216]. Exosomal HSP70 and HSP60 have been associated with disease dissemination, treatment response, and clinical status, although prospective validation remains limited. Major technical barriers include heterogeneity in extracellular‐vesicle isolation, normalization, quantification, and cross‐platform reproducibility [216, 217].

HSPs may also function as predictive or pharmacodynamic biomarkers for HSP‐targeted treatment. Baseline expression of HSP90 clients, including HER2, ALK, EGFR, and RAF–MEK–AKT pathway proteins, has been examined as a predictor of response, but no broadly validated biomarker has emerged [218, 219]. Pharmacodynamic monitoring of HSP70 induction and HSP90‐client depletion may provide more direct evidence of target engagement. Molecular contexts associated with strong HSP90 dependence, such as BRAF^V600E, may define responsive subgroups because mutant BRAF forms a stable HSP90–CDC37 client complex [208, 220].

Circulating and exosomal HSPs can be measured using ELISA, electrochemiluminescence, bead‐based assays, mass spectrometry, and proximity extension assays [47, 215]. Digital ELISA and single‐molecule or single‐vesicle platforms may improve detection of low‐abundance HSP species [47, 221]. Nevertheless, clinical implementation requires reference standards, harmonized pre‐analytical procedures, prospective multicenter cohorts, independent validation, and clarification of whether circulating HSPs arise through active extracellular‐vesicle secretion or passive release from damaged cells [215, 221].

### 3.17 Next‐Generation Therapeutic Modalities: AI, Structural Biology, and Novel Agents

Although HSP90 inhibitors have achieved clinical activity in selected settings, toxicity, adaptive heat‐shock responses, and drug resistance remain important limitations [222]. Computational approaches may assist the identification of cryptic or allosteric binding sites, particularly in HSP70, which lacks a conventional deep hydrophobic pocket [223]. AI‐assisted screening may also support the design of isoform‐selective HSP90 inhibitors and proteolysis‐targeting chimeras that degrade specific oncogenic clients rather than globally suppressing HSP90 activity [224]. Deep‐learning‐enabled virtual screening platforms can accelerate the interrogation of large chemical libraries, although experimentally confirmed HSP‐specific applications remain comparatively limited [225].

Structural studies provide a complementary route to selective targeting. Cryo‐EM analyses of HSP90–co‐chaperone–client complexes have clarified how HSP90–CDC37 organizes kinase clients and revealed potentially druggable protein–protein interfaces [21]. Pharmacological disruption of this interaction, including by DCZ3112 in HER2‐positive breast cancer models, supports the feasibility of targeting chaperone–co‐chaperone interfaces rather than the conserved ATP‐binding pocket [52, 220].

Cryo‐EM has also resolved open, intermediate, and closed conformations of HSP60‐containing chaperonin complexes, providing insight into ATP‐dependent substrate recognition and encapsulation [226]. Recent structures of human HSP60–HSP10 assemblies have revealed nucleotide‐dependent ring organization and asymmetric apical‐domain conformations involved in client engagement [227]. These findings suggest that conformationally defined interfaces within the HSP60–HSP10 cycle may provide targets for mechanism‐based drug development [169]. The ability of KHS101 to disrupt HSP60‐dependent mitochondrial metabolism and inhibit glioblastoma patient‐derived xenografts further supports the therapeutic relevance of this pathway, although KHS101 was not developed through cryo‐EM‐guided design [191].

Antibody‐based strategies may be particularly applicable to membrane‐associated or extracellular HSPs. Membrane HSP70 is expressed by several malignant cell types and can be targeted by the cmHsp70.1 monoclonal antibody, which induces antibody‐dependent cellular cytotoxicity in preclinical models [228, 229]. The anti‐extracellular‐HSP90α antibody HH01 inhibits HSP90α–CD91/LRP1 signaling and reduces invasion and tumor growth in pancreatic cancer models [229, 230].

HSP70‐ and HSP60‐directed small molecules remain largely preclinical [168]. The allosteric HSP70 inhibitor JG‐98 enhances enzalutamide sensitivity through STUB1/CHIP‐associated mechanisms in antiandrogen‐resistant prostate cancer models [231]. Fragment‐based screening and DNA‐encoded libraries may expand the chemical space for selective HSP70 ligands, although validated candidates remain limited [21, 232]. For HSP60, DCEM1 induces HSP60‐associated mitochondrial stress and suppresses prostate cancer growth in preclinical models [233]. Further evaluation should establish the selectivity, pharmacokinetics, normal‐tissue toxicity, and combination potential of these emerging agents.

### 3.18 Rational Combinatorial Strategies: Immunotherapy and Metabolic Checkpoint Blockade

The complexity of tumor metabolism necessitates combinatorial approaches targeting multiple pathways. Co‐targeting HSP inhibitors with glycolytic modulators may synergistically impair tumor energy supply—for instance, combining HSP90 inhibitors with hexokinase or lactate dehydrogenase blockers could disrupt metabolic adaptation [105, 106]. Moreover, the integration of HSP inhibition with immunotherapies, particularly immune checkpoint blockade, represents a highly compelling avenue [107]. Preliminary evidence suggests HSP90 inhibition can enhance tumor immunogenicity by promoting antigen presentation and reducing immunosuppressive cytokines, and this potential likely extends to other HSPs. Similarly, co‐targeting HSPs and specific metabolic checkpoints may induce synthetic lethality and overcome metabolic plasticity [108]. Emerging evidence of HSP involvement in immune evasion—such as HSP70‐mediated PD‐L1 stabilization and GRP78‐driven immunosuppressive signaling—also supports combination with immunotherapy. Future studies should explore optimal sequencing, dosing, and predictive biomarkers for such combinations.

### 3.19 HSP‐Directed Immunotherapies: CAR‐T Cells, Monoclonal Antibodies, and Cancer Vaccines

HSPs can function as direct tumor‐associated targets or as carriers of immunogenic tumor peptides. HSP‐based vaccines, particularly autologous gp96/HSPPC‐96 peptide complexes, have entered clinical evaluation, whereas HSP‐directed CAR‐T‐cell strategies remain largely preclinical [234, 235].

Cell‐surface GRP78/HSPA5 has been explored as a CAR‐T‐cell target because this endoplasmic‐reticulum chaperone can relocalize to the plasma membrane of stressed cancer cells. GRP78‐directed CAR‐T cells have demonstrated antigen‐dependent cytotoxicity in models of acute myeloid leukemia and selected solid and brain tumors [236, 237]. Membrane HSP70 is also expressed by several solid tumors, and CAR‐T cells derived from the cmHsp70.1 recognition module can induce granzyme B and IFN‐γ release against HSP70‐positive tumor cells [238]. The principal concerns are heterogeneous antigen expression, reduced T‐cell trafficking and persistence, and potential recognition of stressed normal tissues.

HSP60 has been proposed as an extracellular or cell‐surface therapeutic target, although the evidence is less developed than that for membrane HSP70 or extracellular HSP90 [22]. Current evidence for anti‐HSP60 antibodies mediating robust antibody‐dependent or complement‐dependent cytotoxicity remains limited, and no anti‐HSP60 monoclonal antibody has entered clinical testing [168, 239]. Further development requires confirmation of tumor‐selective surface expression, careful epitope selection, Fc optimization, and systematic normal‐tissue safety assessment.

HSP–peptide complexes promote antigen cross‐presentation through receptors such as CD91. HSPPC‐96, an autologous tumor‐derived gp96/GRP94–peptide vaccine, has been evaluated in renal cell carcinoma, glioma, melanoma, colorectal cancer, and other malignancies [234, 240]. These vaccines have generally shown acceptable tolerability, but clinical efficacy has been variable. A phase III renal cell carcinoma study did not improve recurrence‐free survival in the overall population, and early glioblastoma studies have not established a definitive survival benefit [235]. Dependence on sufficient patient‐derived tumor tissue also limits scalability.

Most HSP‐directed immunotherapies therefore remain investigational. Target selection and therapeutic modality should be adapted to cancer type and antigen distribution [237, 238]. Antigen escape, intratumoral heterogeneity, on‐target/off‐tumor toxicity, and the immunosuppressive microenvironment remain major obstacles, particularly for CAR‐T therapy in solid tumors [241]. Future studies should prioritize normal‐tissue antigen mapping, biomarker‐guided enrollment, optimized CAR and antibody engineering, and rational combinations with checkpoint blockade or conventional treatments.

### 3.20 Personalized Medicine Approaches Based on HSP Profiling

Tumor genomics, metabolomics, single‐cell technologies, and functional drug screening may identify tumor‐specific HSP expression patterns, client‐protein dependencies, metabolic states, and rational therapeutic combinations [242, 243]. Such multidimensional profiling may be more informative than total HSP expression alone.

A central challenge is the context‐dependent function of individual HSPs. HSP60/HSPD1, for example, may promote or suppress tumor progression depending on cancer lineage, cellular state, subcellular localization, client repertoire, and posttranslational modification [168]. Clarifying these determinants will be essential for distinguishing actionable HSP dependencies from noncausal expression changes.

HSP‐supported metabolic adaptation provides potential therapeutic and biomarker opportunities, but successful precision treatment will require integration of HSP isoform activity, co‐chaperone networks, client‐protein dependence, metabolic phenotype, and adaptive stress responses [9]. These parameters should be prospectively evaluated to identify patients likely to benefit from HSP‐targeted or metabolism‐directed therapies while limiting toxicity in nonresponsive patients.

## 4 Conclusion

HSPs couple proteostasis to cancer metabolic reprogramming by regulating the folding, stability, localization, and activity of metabolic enzymes, nutrient transporters, signaling proteins, and mitochondrial components. The ATP‐dependent conformational cycles of HSP90 and HSP70, the mitochondrial chaperonin activity of HSP60, the client‐selecting functions of HSP40, the nucleotide‐exchange activity of HSP110, and the ATP‐independent holdase functions of small HSPs collectively influence glycolysis, oxidative phosphorylation, lipid and biosynthetic metabolism, redox homeostasis, and metabolic adaptation. Their effects are determined by client specificity, co‐chaperone networks, subcellular localization, and tumor context.

These mechanistic dependencies provide opportunities for pharmacological inhibition, disruption of HSP–client or HSP–co‐chaperone interactions, immune‐based targeting, biomarker development, and rational combination therapy. However, the clinical experience with HSP inhibitors demonstrates that broad suppression of essential chaperone systems is limited by normal‐tissue toxicity, adaptive heat‐shock responses, tumor heterogeneity, drug resistance, and insufficient patient selection. Future therapeutic development should therefore prioritize isoform‐, compartment‐, and client‐selective approaches supported by validated pharmacodynamic and predictive biomarkers.

By integrating HSP structural biology with pathway‐level metabolic regulation, preclinical evidence, and clinical outcomes, this review provides a structure–client–metabolism framework for interpreting the context‐dependent functions of HSPs in cancer. This framework identifies the principal requirements for advancing the field: rigorous distinction between direct and secondary metabolic effects, validation in clinically representative models, characterization of adaptive resistance, development of standardized biomarkers, and prospective evaluation of selective HSP‐targeted strategies in molecularly stratified patient populations.

## Author Contributions

**Sheng Ma**: writing – original draft, investigation, data curation. **Chen‐Qian Liu**: writing – original draft, data curation. **Siyang Ma**: software, formal analysis. **Shao‐Gang Wang**: writing – review and editing, supervision. **Qi‐Dong Xia**: writing – review and editing, supervision, resources. **Meng‐Yao Xu**: funding acquisition, writing – review and editing, supervision, data curation. All authors have read and approved the final manuscript.

## Ethics Statement

The authors have nothing to report.

## Conflicts of Interest

The authors declare no conflicts of interest.

## Data Availability

The authors have nothing to report.

## References

[1] L. Xia , L. Oyang , J. Lin , et al., “The Cancer Metabolic Reprogramming and Immune Response,” Molecular Cancer 20, no. 1 (2021): 28. 33546704 10.1186/s12943-021-01316-8 PMC7863491

[2] B. Faubert , A. Solmonson , and R. J. DeBerardinis , “Metabolic Reprogramming and Cancer Progression,” Science (New York, NY) 368, no. 6487 (2020): 368. 10.1126/science.aaw5473 PMC7227780 32273439

[3] W. H. Koppenol , P. L. Bounds , and C. V. Dang , “Otto Warburg's Contributions to Current Concepts of Cancer Metabolism,” Nature Reviews Cancer 11, no. 5 (2011): 325–337. 21508971 10.1038/nrc3038

[4] K. Hachani , M. Ghanem , A. G. Pockley , et al., “Heat Shock Protein 70 (Hsp70) as a Target for Advancing Immunotherapy in Solid Tumors,” Cytokine & Growth Factor Reviews: 86 (2025): 83–95. 40947322 10.1016/j.cytogfr.2025.09.002

[5] I. Skrabalak , A. Rajtak , B. Malachowska , et al., “Therapy Resistance: Modulating Evolutionarily Conserved Heat Shock Protein Machinery in Cancer,” Cancer Letters 616 (2025): 217571. 39986370 10.1016/j.canlet.2025.217571

[6] S. M. Fendt , “100 Years of the Warburg Effect: A Cancer Metabolism Endeavor,” Cell 187, no. 15 (2024): 3824–3828. 39059359 10.1016/j.cell.2024.06.026

[7] D. Hanahan , “Hallmarks of Cancer: New Dimensions,” Cancer Discovery 12, no. 1 (2022): 31–46. 35022204 10.1158/2159-8290.CD-21-1059

[8] W. F. Zuo , Q. Pang , X. Zhu , et al., “Heat Shock Proteins as Hallmarks of Cancer: Insights From Molecular Mechanisms to Therapeutic Strategies,” Journal of Hematology & Oncology 17, no. 1 (2024): 81. 39232809 10.1186/s13045-024-01601-1 PMC11375894

[9] W. F. Zuo , Q. Pang , X. Zhu , et al., “Heat Shock Proteins as Hallmarks of Cancer: Insights From Molecular Mechanisms to Therapeutic Strategies,” Journal of Hematology & Oncology 17, no. 1 (2024): 81. 39232809 10.1186/s13045-024-01601-1 PMC11375894

[10] G. Chakafana and A. Shonhai , “The Role of Non‐Canonical Hsp70s (Hsp110/Grp170) in Cancer,” Cells 10, no. 2 (2021):254. 33525518 10.3390/cells10020254 PMC7911927

[11] C. Prodromou and D. M. Bjorklund , “Advances Towards Understanding the Mechanism of Action of the Hsp90 Complex,” Biomolecules 12, no. 5 (2022): 600. 35625528 10.3390/biom12050600 PMC9138868

[12] Y. Hu , J. Li , H. Chen , et al., “Autophagy Related 5 Promotes Mitochondrial Fission and Inflammation via HSP90‐HIF‐1α‐Mediated Glycolysis in Kidney Fibrosis,” Advanced Science (Weinheim, Baden‐Wurttemberg, Germany) 12, no. 17 (2025): e2414673. 40047327 10.1002/advs.202414673 PMC12061336

[13] G. Shen , S. Liu , Y. Cao , et al., “HSP90 Co‐Regulates the Formation and Nuclear Distribution of the Glycolytic Output Complex to Promote Resistance and Poor Prognosis in Gastric Cancer Patients,” Journal of Translational Medicine 23, no. 1 (2025): 172. 39930487 10.1186/s12967-025-06196-w PMC11812214

[14] A. J. Ambrose and E. Chapman , “Function, Therapeutic Potential, and Inhibition of Hsp70 Chaperones,” Journal of Medicinal Chemistry 64, no. 11 (2021): 7060–7082. 34009983 10.1021/acs.jmedchem.0c02091

[15] G. Sha , Z. Jiang , W. Zhang , et al., “The Multifunction of HSP70 in Cancer: Guardian or Traitor to the Survival of Tumor Cells and the next Potential Therapeutic Target,” International Immunopharmacology 122 (2023): 110492. 37390645 10.1016/j.intimp.2023.110492

[16] H. Dong , Y. Xia , J. Qi , et al., “lncRNA IGFL2‐AS1 Mediates NSCLC Chemoresistance via YBX1‐Induced HSPA1A/RAP1 Activation,” Cellular & Molecular Biology Letters 30, no. 1 (2025): 133. 41204108 10.1186/s11658-025-00808-5 PMC12595904

[17] M. A. Vostakolaei , L. Hatami‐Baroogh , G. Babaei , et al., “Hsp70 in Cancer: A Double Agent in the Battle Between Survival and Death,” Journal of Cellular Physiology 236, no. 5 (2021): 3420–3444. 33169384 10.1002/jcp.30132

[18] M. Haslbeck and E. Vierling , “A First Line of Stress Defense: Small Heat Shock Proteins and Their Function in Protein Homeostasis,” Journal of Molecular Biology 427, no. 7 (2015): 1537–1548. 25681016 10.1016/j.jmb.2015.02.002 PMC4360138

[19] T. de Los Reyes and S. Casas‐Tintó , “Neural Functions of Small Heat Shock Proteins,” Neural Regeneration Research 17, no. 3 (2022): 512–515. 34380880 10.4103/1673-5374.320975 PMC8504394

[20] R. Rosenzweig , N. B. Nillegoda , M. P. Mayer , et al., “The Hsp70 Chaperone Network,” Nature Reviews Molecular Cell Biology 20, no. 11 (2019): 665–680. 31253954 10.1038/s41580-019-0133-3

[21] A. Wentink , R. Rosenzweig , H. Kampinga , et al., “Mechanisms and Regulation of the Hsp70 Chaperone Network,” Nature Reviews Molecular Cell Biology 27, no. 2 (2026): 110–128. 41145833 10.1038/s41580-025-00890-9

[22] Y. Tang , Y. Zhou , S. Fan , et al., “The Multiple Roles and Therapeutic Potential of HSP60 in Cancer,” Biochemical Pharmacology 201 (2022): 115096. 35609646 10.1016/j.bcp.2022.115096

[23] L. Torielli , F. Guarra , H. Shao , et al., “Pathogenic Mutation Impairs Functional Dynamics of Hsp60 in Mono‐ and Oligomeric States,” Nature Communications 16, no. 1 (2025): 3158. 10.1038/s41467-025-57958-5 PMC11968893 40180932

[24] C. Zhou , H. Sun , C. Zheng , et al., “Oncogenic HSP60 Regulates Mitochondrial Oxidative Phosphorylation to Support Erk1/2 Activation During Pancreatic Cancer Cell Growth,” Cell Death & Disease 9, no. 2 (2018): 161. 29415987 10.1038/s41419-017-0196-z PMC5833694

[25] J. A. Woytash , R. Kumar , A. K. Chaudhary , et al., “Mitochondrial Unfolded Protein Response‐Dependent β‐Catenin Signaling Promotes Neuroendocrine Prostate Cancer,” Oncogene 44, no. 12 (2025): 820–834. 39690273 10.1038/s41388-024-03261-4 PMC12314736

[26] Y. Jiang , P. Rossi , and C. G. Kalodimos , “Structural Basis for Client Recognition and Activity of Hsp40 Chaperones,” Science (New York, NY) 365, no. 6459 (2019): 1313–1319. 10.1126/science.aax1280 PMC7023980 31604242

[27] M. M. Biebl , F. Delhommel , O. Faust , et al., “NudC Guides Client Transfer Between the Hsp40/70 and Hsp90 Chaperone Systems,” Molecular Cell 82, no. 3 (2022): 555–569. 35063133 10.1016/j.molcel.2021.12.031

[28] B. Wang and M. R. Pratt , “Potential for Targeting Small Heat Shock Protein Modifications,” Trends in Pharmacological Sciences 45, no. 7 (2024): 583–585. 38704305 10.1016/j.tips.2024.04.002 PMC11227382

[29] K. Reinle , A. Mogk , and B. Bukau , “The Diverse Functions of Small Heat Shock Proteins in the Proteostasis Network,” Journal of Molecular Biology 434, no. 1 (2022): 167157. 34271010 10.1016/j.jmb.2021.167157

[30] H. H. Kampinga , J. Hageman , M. J. Vos , et al., “Guidelines for the Nomenclature of the Human Heat Shock Proteins,” Cell Stress & Chaperones 14, no. 1 (2009): 105–111. 18663603 10.1007/s12192-008-0068-7 PMC2673902

[31] C. N. Woods , M. K. Janowska , L. D. Ulmer , et al., “Activation Mechanism of Small Heat Shock Protein HSPB5 Revealed by Disease‐Associated Mutants,” Proceedings of the National Academy of Sciences of the United States of America 122, no. 20 (2025): e2425061122. 40377988 10.1073/pnas.2425061122 PMC12107100

[32] Y. X. Jiang , J. Xu , Z. Y. Wang , et al., “HSP27 Promotes Cutaneous Squamous Cell Carcinoma Progression by Inhibiting Ferroptosis,” BioFactors (Oxford, England) 52, no. 1 (2026): e70073. 41482717 10.1002/biof.70073

[33] R. Shan , N. Liu , Y. Yan , et al., “Apoptosis, Autophagy and Atherosclerosis: Relationships and the Role of Hsp27,” Pharmacological Research 166 (2021): 105169. 33053445 10.1016/j.phrs.2020.105169

[34] S. Lu , J. Hu , O. A. Arogundade , et al., “Heat‐shock Chaperone HSPB1 Regulates Cytoplasmic TDP‐43 Phase Separation and Liquid‐to‐Gel Transition,” Nature Cell Biology 24, no. 9 (2022): 1378–1393. 36075972 10.1038/s41556-022-00988-8 PMC9872726

[35] M. Bsibsi , L. A. Peferoen , I. R. Holtman , et al., “Demyelination During Multiple Sclerosis Is Associated With Combined Activation of Microglia/Macrophages by IFN‐γ and Alpha B‐crystallin,” Acta Neuropathologica 128, no. 2 (2014): 215–229. 24997049 10.1007/s00401-014-1317-8

[36] J. V. Moyano , J. R. Evans , F. Chen , et al., “AlphaB‐crystallin Is a Novel Oncoprotein That Predicts Poor Clinical Outcome in Breast Cancer,” Journal of Clinical Investigation 116, no. 1 (2006): 261–270. 16395408 10.1172/JCI25888 PMC1323258

[37] J. Xu , Y. Guo , W. Ning , et al., “Comprehensive Analysis of Heat Shock Proteins in Glioma Revealed the Association With Glioma‐Associated Myeloid Cells,” Genes and Immunity 26, no. 3 (2025): 200–212. 40234584 10.1038/s41435-025-00327-5 PMC12170335

[38] Y. Feng , Z. Huang , F. Lu , et al., “‐Br‐cGMP Activates HSPB6 and Increases the Antineoplastic Activity of Quinidine in Prostate Cancer,” Cell Death Discovery 10, no. 1 (2024): 90. 38374143 10.1038/s41420-024-01853-3 PMC10876707

[39] Y. Cao , Y. Yu , T. Tian , et al., “HSPB6 based on Integrative Multi‐Omics Analysis Can Suppress Colorectal Cancer Progression by Downregulating HSPA6 Expression Through the JNK‐JUND Axis,” Life Sciences 381 (2025): 124001. 41043501 10.1016/j.lfs.2025.124001

[40] M. K. Janowska , H. E. R. Baughman , C. N. Woods , et al., “Mechanisms of Small Heat Shock Proteins,” Cold Spring Harbor Perspectives in Biology 11, no. 10 (2019): a034025. 30833458 10.1101/cshperspect.a034025 PMC6771367

[41] S. K. Calderwood and J. Gong , “Heat Shock Proteins Promote Cancer: It's a Protection Racket,” Trends in Biochemical Sciences 41, no. 4 (2016): 311–323. 26874923 10.1016/j.tibs.2016.01.003 PMC4911230

[42] S. K. Calderwood , M. A. Khaleque , D. B. Sawyer , et al., “Heat Shock Proteins in Cancer: Chaperones of Tumorigenesis,” Trends in Biochemical Sciences 31, no. 3 (2006): 164–172. 16483782 10.1016/j.tibs.2006.01.006

[43] A. E. Archer , A. T. Von Schulze , and P. C. Geiger , “Exercise, Heat Shock Proteins and Insulin Resistance,” Philosophical Transactions of the Royal Society of London Series B, Biological sciences 373 (2018): 1738. 10.1098/rstb.2016.0529 PMC5717529 29203714

[44] F. I. Yapici , E. Seidel , A. Dahlhaus , et al., “An Atlas of Ferroptosis‐Induced Secretomes,” Cell Death and Differentiation 32, no. 11 (2025): 1986–2008. 40281125 10.1038/s41418-025-01517-4 PMC12572367

[45] B. Parma , H. Wurdak , and P. Ceppi , “Harnessing Mitochondrial Metabolism and Drug Resistance in Non‐Small Cell Lung Cancer and Beyond by Blocking Heat‐Shock Proteins,” Drug Resistance Updates 65 (2022): 100888. 36332495 10.1016/j.drup.2022.100888

[46] J. Wu , T. Liu , Z. Rios , et al., “Heat Shock Proteins and Cancer,” Trends in Pharmacological Sciences 38, no. 3 (2017): 226–256. 28012700 10.1016/j.tips.2016.11.009

[47] M. Regimbeau , J. Abrey , V. Vautrot , et al., “Heat Shock Proteins and Exosomes in Cancer Theranostics,” Seminars in Cancer Biology 86, no. Pt 1 (2022): 46–57. 10.1016/j.semcancer.2021.07.014 34343652

[48] J. P. Schuermann , J. Jiang , J. Cuellar , et al., “Structure of the Hsp110:Hsc70 Nucleotide Exchange Machine,” Molecular Cell 31, no. 2 (2008): 232–243. 18550409 10.1016/j.molcel.2008.05.006 PMC2892728

[49] S. Leone , A. Srivastava , A. Herrero‐Ruiz , et al., “HSP70 Binds to Specific Non‐Coding RNA and Regulates Human RNA Polymerase III,” Molecular Cell 84, no. 4 (2024): 687–701.e7. 38266641 10.1016/j.molcel.2024.01.001

[50] S. Polier , Z. Dragovic , F. U. Hartl , et al., “Structural Basis for the Cooperation of Hsp70 and Hsp110 Chaperones in Protein Folding,” Cell 133, no. 6 (2008): 1068–1079. 18555782 10.1016/j.cell.2008.05.022

[51] M. Mauthe , N. van de Beek , M. Mari , et al., “A Chaperone‐Proteasome‐Based Fragmentation Machinery Is Essential for Aggrephagy,” Nature Cell Biology 27, no. 9 (2025): 1448–1464. 40866512 10.1038/s41556-025-01747-1 PMC12431860

[52] K. A. Verba , R. Y. Wang , A. Arakawa , et al., “Atomic Structure of Hsp90‐Cdc37‐Cdk4 Reveals That Hsp90 Traps and Stabilizes an Unfolded Kinase,” Science (New York, NY) 352, no. 6293 (2016): 1542–1547. 10.1126/science.aaf5023 PMC5373496 27339980

[53] B. Ou , H. Sun , J. Zhao , et al., “Polo‐Like Kinase 3 Inhibits Glucose Metabolism in Colorectal Cancer by Targeting HSP90/STAT3/HK2 Signaling,” Journal of Experimental & Clinical Cancer Research 38, no. 1 (2019): 426. 31655629 10.1186/s13046-019-1418-2 PMC6815449

[54] Z. Albakova , G. A. Armeev , L. M. Kanevskiy , et al., “HSP70 Multi‐Functionality in Cancer,” Cells 9, no. 3 (2020): 587. 32121660 10.3390/cells9030587 PMC7140411

[55] A. S. Lee , “Glucose‐Regulated Proteins in Cancer: Molecular Mechanisms and Therapeutic Potential,” Nature Reviews Cancer 14, no. 4 (2014): 263–276. 24658275 10.1038/nrc3701 PMC4158750

[56] Q. Wang , L. Li , X. Gao , et al., “Targeting GRP75 With a Chlorpromazine Derivative Inhibits Endometrial Cancer Progression Through GRP75‐IP3R‐Ca(2+)‐AMPK Axis,” Advanced Science (Weinheim, Baden‐Wurttemberg, Germany) 11, no. 15 (2024): e2304203. 38342610 10.1002/advs.202304203 PMC11022737

[57] C. S. Shin , S. Meng , S. D. Garbis , et al., “LONP1 and mtHSP70 Cooperate to Promote Mitochondrial Protein Folding,” Nature Communications 12, no. 1 (2021): 265. 10.1038/s41467-020-20597-z PMC7801493 33431889

[58] Y. Liu , H. Shi , C. Li , et al., “Dusp15 Modulates mtHsp70 Thr116 Phosphorylation State to Preserve Mito‐UPR and Attenuate Cardiac Dysfunction in Diabetic Cardiomyopathy,” Cardiovascular Diabetology 25, no. 1 (2026): 98. 41794713 10.1186/s12933-026-03125-z PMC13020309

[59] C. Qian , C. Hu , Y. Xu , et al., “Intestinal Epithelial‐Derived USP13 Alleviates Colonic Inflammation by Suppressing GRP78‐mediated Endoplasmic Reticulum Stress,” Advanced Science (Weinheim, Baden‐Wurttemberg, Germany) 12, no. 38 (2025): e00741. 40679368 10.1002/advs.202500741 PMC12520541

[60] M. Y. Cheng , F. U. Hartl , and A. L. Horwich , “The Mitochondrial Chaperonin hsp60 Is Required for Its Own Assembly,” Nature 348, no. 6300 (1990): 455–458. 1978929 10.1038/348455a0

[61] M. Jung , M. Kim , S. J. Ham , et al., “In Situ Characterization of Mitochondrial Hsp60‐Hsp10 Chaperone Complex Under Folding Stress,” Science Advances 11, no. 43 (2025): eadw6064. 41124257 10.1126/sciadv.adw6064 PMC12542933

[62] M. Morgenstern , C. D. Peikert , P. Lübbert , et al., “Quantitative High‐Confidence Human Mitochondrial Proteome and Its Dynamics in Cellular Context,” Cell Metabolism 33, no. 12 (2021): 2464–2483.e18. 34800366 10.1016/j.cmet.2021.11.001 PMC8664129

[63] Y. Shi , Z. Wu , S. Liu , et al., “Targeting PRMT3 Impairs Methylation and Oligomerization of HSP60 to Boost Anti‐Tumor Immunity by Activating cGAS/STING Signaling,” Nature Communications 15, no. 1 (2024): 7930. 10.1038/s41467-024-52170-3 PMC11387718 39256398

[64] B. Choi , M. Choi , C. Park , et al., “Cytosolic Hsp60 Orchestrates the Survival and Inflammatory Responses of Vascular Smooth Muscle Cells in Injured Aortic Vessels,” Cardiovascular Research 106, no. 3 (2015): 498–508. 25870185 10.1093/cvr/cvv130

[65] K. Chen , Y. Zhang , H. Ruan , et al., “PRKAB2 as a Tumor Suppressor in Renal Cell Carcinoma: Inhibiting Mitophagy via the LRPPRC‐PRKN/Parkin Interaction and Cardiolipin Biosynthesis,” Autophagy 22, no. 5 (2026): 982–1002. 41612594 10.1080/15548627.2026.2623985 PMC13232994

[66] O. Faust , M. Abayev‐Avraham , A. S. Wentink , et al., “HSP40 Proteins Use Class‐Specific Regulation to Drive HSP70 Functional Diversity,” Nature 587, no. 7834 (2020): 489–494. 33177718 10.1038/s41586-020-2906-4

[67] Y. Jiang , Z. Ibrahim , Y. Xia , et al., “Mechanisms of Assembly and Function of the Hsp70‐Hsp40 Chaperone Machinery,” Molecular Cell 85, no. 21 (2025): 4032–4046. 41092901 10.1016/j.molcel.2025.09.023 PMC12677917

[68] T. S. Voegeli and R. W. Currie , “siRNA Knocks Down Hsp27 and Increases Angiotensin II‐Induced Phosphorylated NF‐kappaB p65 Levels in Aortic Smooth Muscle Cells,” Inflammation Research 58, no. 6 (2009): 336–343. 19247578 10.1007/s00011-009-8166-2

[69] A. Burban , A. Sharanek , C. Guguen‐Guillouzo , et al., “Endoplasmic Reticulum Stress Precedes Oxidative Stress in Antibiotic‐Induced Cholestasis and Cytotoxicity in Human Hepatocytes,” Free Radical Biology & Medicine 115 (2018): 166–178. 29191461 10.1016/j.freeradbiomed.2017.11.017

[70] A. J. Macario and E. C. de Macario , “Molecular Mechanisms in Chaperonopathies: Clues to Understanding the Histopathological Abnormalities and Developing Novel Therapies,” Journal of Pathology 250, no. 1 (2020): 9–18. 31579936 10.1002/path.5349

[71] R. Teng , Z. Liu , H. Tang , et al., “HSP60 Silencing Promotes Warburg‐Like Phenotypes and Switches the Mitochondrial Function From ATP Production to Biosynthesis in ccRCC Cells,” Redox Biology 24 (2019): 101218. 31112866 10.1016/j.redox.2019.101218 PMC6526248

[72] P. S. Wu , Y. H. Chang , and C. C. Pan , “High Expression of Heat Shock Proteins and Heat Shock Factor‐1 Distinguishes an Aggressive Subset of Clear Cell Renal Cell Carcinoma,” Histopathology 71, no. 5 (2017): 711–718. 28617974 10.1111/his.13284

[73] V. P. Tan and S. Miyamoto , “HK2/Hexokinase‐II Integrates Glycolysis and Autophagy to Confer Cellular Protection,” Autophagy 11, no. 6 (2015): 963–964. 26075878 10.1080/15548627.2015.1042195 PMC4502787

[74] S. P. Mathupala , Y. H. Ko , and P. L. Pedersen , “Hexokinase‐2 Bound to Mitochondria: Cancer's Stygian Link to the “Warburg Effect” and a Pivotal Target for Effective Therapy,” Seminars in Cancer Biology 19, no. 1 (2009): 17–24. 19101634 10.1016/j.semcancer.2008.11.006 PMC2714668

[75] S. P. Mathupala , Y. H. Ko , and P. L. Pedersen , “Hexokinase II: Cancer's Double‐Edged Sword Acting as both Facilitator and Gatekeeper of Malignancy When Bound to Mitochondria,” Oncogene 25, no. 34 (2006): 4777–4786. 16892090 10.1038/sj.onc.1209603 PMC3385868

[76] P. Picone , D. Nuzzo , L. Caruana , et al., “Curcumin Induces Apoptosis in Human Neuroblastoma Cells via Inhibition of AKT and Foxo3a Nuclear Translocation,” Free Radical Research 48, no. 12 (2014): 1397–1408. 25179440 10.3109/10715762.2014.960410

[77] Y. Wang , H. Shu , Y. Qu , et al., “PKM2 functions as a Histidine Kinase to Phosphorylate PGAM1 and Increase Glycolysis Shunts in Cancer,” The EMBO Journal 43, no. 12 (2024): 2368–2396. 38750259 10.1038/s44318-024-00110-8 PMC11183095

[78] Z. Fu , M. Deng , Q. Zhou , et al., “Arsenic Activated GLUT1‐mTORC1/HIF‐1α‐PKM2 Positive Feedback Networks Promote Proliferation and Migration of Bladder Epithelial Cells,” Science of the Total Environment 947 (2024): 174538. 38977090 10.1016/j.scitotenv.2024.174538

[79] N. Jing , X. Du , Y. Liang , et al., “PAX6 promotes Neuroendocrine Phenotypes of Prostate Cancer via Enhancing MET/STAT5A‐Mediated Chromatin Accessibility,” Journal of Experimental & Clinical Cancer Research 43, no. 1 (2024): 144. 38745318 10.1186/s13046-024-03064-1 PMC11094950

[80] J. Q. Xu , Y. L. Fu , J. Zhang , et al., “Targeting Glycolysis in Non‐small Cell Lung Cancer: Promises and Challenges,” Frontiers in Pharmacology 13 (2022): 1037341. 36532721 10.3389/fphar.2022.1037341 PMC9748442

[81] X. Wu , J. Guo , Y. Chen , et al., “The 60‐kDa Heat Shock Protein Regulates Energy Rearrangement and Protein Synthesis to Promote Proliferation of Multiple Myeloma Cells,” British Journal of Haematology 190, no. 5 (2020): 741–752. 32155663 10.1111/bjh.16569

[82] Y. Tanaka , T. Hosoyama , A. Mikamo , et al., “Hypoxic Preconditioning of Human Cardiosphere‐Derived Cell Sheets Enhances Cellular Functions via Activation of the PI3K/Akt/mTOR/HIF‐1α Pathway,” American Journal of Translational Research 9, no. 2 (2017): 664–673. 28337294 PMC5340701

[83] X. Hu , H. Li , T. K. Ip , et al., “Arsenic Trioxide Targets Hsp60, Triggering Degradation of p53 and Survivin,” Chemical Science 12, no. 32 (2021): 10893–10900. 34476069 10.1039/d1sc03119h PMC8372542

[84] A. M. Gammazza , C. Campanella , R. Barone , et al., “Doxorubicin Anti‐Tumor Mechanisms Include Hsp60 Post‐Translational Modifications Leading to the Hsp60/p53 Complex Dissociation and Instauration of Replicative Senescence,” Cancer Letters 385 (2017): 75–86. 27836734 10.1016/j.canlet.2016.10.045

[85] K. Bensaad , A. Tsuruta , M. A. Selak , et al., “TIGAR, a p53‐Inducible Regulator of Glycolysis and Apoptosis,” Cell 126, no. 1 (2006): 107–120. 16839880 10.1016/j.cell.2006.05.036

[86] K. M. Lin , B. Lin , I. Y. Lian , et al., “Combined and Individual Mitochondrial HSP60 and HSP10 Expression in Cardiac Myocytes Protects Mitochondrial Function and Prevents Apoptotic Cell Deaths Induced by Simulated Ischemia‐Reoxygenation,” Circulation 103, no. 13 (2001): 1787–1792. 11282911 10.1161/01.cir.103.13.1787

[87] F. A. Dekker and S. G. D. Rüdiger , “The Mitochondrial Hsp90 TRAP1 and Alzheimer's Disease,” Frontiers in Molecular Biosciences 8 (2021): 697913. 34222342 10.3389/fmolb.2021.697913 PMC8249562

[88] A. Joshi , L. Dai , Y. Liu , et al., “The Mitochondrial HSP90 Paralog TRAP1 Forms an OXPHOS‐Regulated Tetramer and Is Involved in Mitochondrial Metabolic Homeostasis,” BMC Biology 18, no. 1 (2020): 10. 31987035 10.1186/s12915-020-0740-7 PMC6986101

[89] S. Purushottam Dharaskar , K. Paithankar , and A. Kanugovi Vijayavittal , “Mitochondrial Chaperone, TRAP1 Modulates Mitochondrial Dynamics and Promotes Tumor Metastasis,” Mitochondrion 54 (2020): 92–101. 32784002 10.1016/j.mito.2020.08.001

[90] F. W. Chen , J. P. Davies , R. Calvo , et al., “Activation of Mitochondrial TRAP1 Stimulates Mitochondria‐Lysosome Crosstalk and Correction of Lysosomal Dysfunction,” Iscience 25, no. 9 (2022): 104941. 36065186 10.1016/j.isci.2022.104941 PMC9440283

[91] J. P. Mandal , C. N. Shiue , Y. C. Chen , et al., “PKCδ Mediates Mitochondrial ROS Generation and Oxidation of HSP60 to Relieve RKIP Inhibition on MAPK Pathway for HCC Progression,” Free Radical Biology & Medicine 163 (2021): 69–87. 33307168 10.1016/j.freeradbiomed.2020.12.003

[92] T. H. Chen , C. K. Chou , and K. M. Lin , “Aberrant Mitochondrial hsp60 Expression Affects Mitochondria Homeostasis and Results in Muscle Dystrophy and Premature Death,” Cell Death & Disease 17, no. 1 (2026): 9. 41507123 10.1038/s41419-025-08260-1 PMC12783612

[93] S. W. Weng , J. C. Wu , F. C. Shen , et al., “Chaperonin Counteracts Diet‐Induced Non‐Alcoholic Fatty Liver Disease by Aiding Sirtuin 3 in the Control of Fatty Acid Oxidation,” Diabetologia 66, no. 5 (2023): 913–930. 36692509 10.1007/s00125-023-05869-9

[94] S. Dai , Z. Tang , J. Cao , et al., “Suppression of the HSF1‐Mediated Proteotoxic Stress Response by the Metabolic Stress Sensor AMPK,” EMBO Journal 34, no. 3 (2015): 275–293. 25425574 10.15252/embj.201489062 PMC4339117

[95] L. Panneerselvam , A. Raghunath , and E. Perumal , “Differential Expression of Myocardial Heat Shock Proteins in Rats Acutely Exposed to Fluoride,” Cell Stress & Chaperones 22, no. 5 (2017): 743–750. 28451878 10.1007/s12192-017-0801-1 PMC5573692

[96] K. H. Su , S. Dai , Z. Tang , et al., “Heat Shock Factor 1 Is a Direct Antagonist of AMP‐Activated Protein Kinase,” Molecular Cell 76, no. 4 (2019): 546–561.e8. 31561952 10.1016/j.molcel.2019.08.021 PMC8170832

[97] M. Boysen , R. Kityk , and M. P. Mayer , “Hsp70‐ and Hsp90‐Mediated Regulation of the Conformation of p53 DNA Binding Domain and p53 Cancer Variants,” Molecular Cell 74, no. 4 (2019): 831–843.e4. 31027880 10.1016/j.molcel.2019.03.032

[98] V. Dahiya , G. Agam , J. Lawatscheck , et al., “Coordinated Conformational Processing of the Tumor Suppressor Protein p53 by the Hsp70 and Hsp90 Chaperone Machineries,” Molecular Cell 74, no. 4 (2019): 816–830.e7. 31027879 10.1016/j.molcel.2019.03.026

[99] W. Neupert and M. Brunner , “The Protein Import Motor of Mitochondria,” Nature Reviews Molecular Cell Biology 3, no. 8 (2002): 555–565. 12154367 10.1038/nrm878

[100] D. G. Hardie , F. A. Ross , and S. A. Hawley , “AMPK: A Nutrient and Energy Sensor That Maintains Energy Homeostasis,” Nature Reviews Molecular Cell Biology 13, no. 4 (2012): 251–262. 22436748 10.1038/nrm3311 PMC5726489

[101] L. Ruan , C. Zhou , E. Jin , et al., “Cytosolic Proteostasis Through Importing of Misfolded Proteins Into Mitochondria,” Nature 543, no. 7645 (2017): 443–446. 28241148 10.1038/nature21695 PMC5793917

[102] X. Tang , C. Chang , M. Hao , et al., “Heat Shock Protein‐90alpha (Hsp90α) Stabilizes Hypoxia‐inducible Factor‐1α (HIF‐1α) in Support of Spermatogenesis and Tumorigenesis,” Cancer Gene Therapy 28, no. 9 (2021): 1058–1070. 33664459 10.1038/s41417-021-00316-6

[103] S. H. Lee , M. Golinska , and J. R. Griffiths , “HIF‐1‐Independent Mechanisms Regulating Metabolic Adaptation in Hypoxic Cancer Cells,” Cells 10, no. 9 (2021): 2371. 34572020 10.3390/cells10092371 PMC8472468

[104] F. Chen , C. Tang , F. Yang , et al., “HSP90 inhibition Suppresses Tumor Glycolytic Flux to Potentiate the Therapeutic Efficacy of Radiotherapy for Head and Neck Cancer,” Science Advances 10, no. 8 (2024): eadk3663. 38394204 10.1126/sciadv.adk3663 PMC10889358

[105] T. Yamaguchi , K. Yanagisawa , R. Sugiyama , et al., “NKX2‐1/TITF1/TTF‐1‐Induced ROR1 Is Required to Sustain EGFR Survival Signaling in Lung Adenocarcinoma,” Cancer Cell 21, no. 3 (2012): 348–361. 22439932 10.1016/j.ccr.2012.02.008

[106] G. Cannino , A. Urbani , M. Gaspari , et al., “The Mitochondrial Chaperone TRAP1 Regulates F‐ATP Synthase Channel Formation,” Cell Death and Differentiation 29, no. 12 (2022): 2335–2346. 35614131 10.1038/s41418-022-01020-0 PMC9751095

[107] S. Wei , D. Yin , S. Yu , et al., “Antitumor Activity of a Mitochondrial‐Targeted HSP90 Inhibitor in Gliomas,” Clinical Cancer Research 28, no. 10 (2022): 2180–2195. 35247901 10.1158/1078-0432.CCR-21-0833 PMC9757132

[108] Y. C. Chae , M. C. Caino , S. Lisanti , et al., “Control of Tumor Bioenergetics and Survival Stress Signaling by Mitochondrial HSP90s,” Cancer Cell 22, no. 3 (2012): 331–344. 22975376 10.1016/j.ccr.2012.07.015 PMC3615709

[109] M. C. Caino , Y. C. Chae , V. Vaira , et al., “Metabolic Stress Regulates Cytoskeletal Dynamics and Metastasis of Cancer Cells,” The Journal of Clinical Investigation 123, no. 7 (2013): 2907–2920. 23921130 10.1172/JCI67841 PMC3998961

[110] V. Dahiya , D. A. Rutz , P. Moessmer , et al., “The Switch From Client Holding to Folding in the Hsp70/Hsp90 Chaperone Machineries Is Regulated by a Direct Interplay Between co‐chaperones,” Molecular Cell 82, no. 8 (2022): 1543–1556.e6. 35176233 10.1016/j.molcel.2022.01.016

[111] K. Bhattacharya and D. Picard , “The Hsp70‐Hsp90 Go‐Between Hop/Stip1/Sti1 Is a Proteostatic Switch and May be a Drug Target in Cancer and Neurodegeneration,” Cellular and Molecular Life Sciences 78, no. 23 (2021): 7257–7273. 34677645 10.1007/s00018-021-03962-z PMC8629791

[112] K. Bhattacharya , L. Weidenauer , T. M. Luengo , et al., “The Hsp70‐Hsp90 Co‐Chaperone Hop/Stip1 Shifts the Proteostatic Balance From Folding towards Degradation,” Nature Communications 11, no. 1 (2020): 5975. 10.1038/s41467-020-19783-w PMC7688965 33239621

[113] T. M. Luengo , M. P. Mayer , and S. G. D. Rüdiger , “The Hsp70‐Hsp90 Chaperone Cascade in Protein Folding,” Trends in Cell Biology 29, no. 2 (2019): 164–177. 30502916 10.1016/j.tcb.2018.10.004

[114] Y. Chin , K. E. Gumilar , X. G. Li , et al., “Targeting HSF1 for Cancer Treatment: Mechanisms and Inhibitor Development,” Theranostics 13, no. 7 (2023): 2281–2300. 37153737 10.7150/thno.82431 PMC10157728

[115] Y. Liu , C. Li , H. Liu , et al., “Combination Therapy Involving HSP90 Inhibitors for Combating Cancer: An Overview of Clinical and Preclinical Progress,” Archives of Pharmacal Research 47, no. 5 (2024): 442–464. 38632167 10.1007/s12272-024-01494-1

[116] A. S. Bie , C. Cömert , R. Körner , et al., “An Inventory of Interactors of the Human HSP60/HSP10 Chaperonin in the Mitochondrial Matrix Space,” Cell Stress & Chaperones 25, no. 3 (2020): 407–416. 32060690 10.1007/s12192-020-01080-6 PMC7192978

[117] T. J. Corydon , J. Hansen , P. Bross , et al., “Down‐Regulation of Hsp60 Expression by RNAi Impairs Folding of Medium‐chain Acyl‐CoA Dehydrogenase Wild‐Type and Disease‐Associated Proteins,” Molecular Genetics and Metabolism 85, no. 4 (2005): 260–270. 15927499 10.1016/j.ymgme.2005.04.003

[118] S. K. Tan , H. Y. Hougen , J. R. Merchan , et al., “Fatty Acid Metabolism Reprogramming in ccRCC: Mechanisms and Potential Targets,” Nature Reviews Urology 20, no. 1 (2023): 48–60. 36192502 10.1038/s41585-022-00654-6 PMC10826284

[119] M. K. Singh , Y. Shin , S. Han , et al., “Molecular Chaperonin HSP60: Current Understanding and Future Prospects,” International Journal of Molecular Sciences 25, no. 10 (2024): 5483. 38791521 10.3390/ijms25105483 PMC11121636

[120] S. Li , H. Yuan , L. Li , et al., “Oxidative Stress and Reprogramming of Lipid Metabolism in Cancers,” Antioxidants (Basel, Switzerland) 14, no. 2 (2025): 201. 40002387 10.3390/antiox14020201 PMC11851681

[121] J. Lin , D. Rao , M. Zhang , et al., “Metabolic Reprogramming in the Tumor Microenvironment of Liver Cancer,” Journal of Hematology & Oncology 17, no. 1 (2024): 6. 38297372 10.1186/s13045-024-01527-8 PMC10832230

[122] S. Nong , X. Han , Y. Xiang , et al., “Metabolic Reprogramming in Cancer: Mechanisms and Therapeutics,” MedComm 4, no. 2 (2023): e218. 36994237 10.1002/mco2.218 PMC10041388

[123] M. Y. Xu , S. Ma , S. Y. Ma , et al., “Targeting the HSP60/p53 Axis With Extracellular Vesicle‐Delivered siRNA Reprograms Glycolysis in Prostate Cancer,” International Journal of Biological Sciences 22, no. 2 (2026): 641–662. 41522350 10.7150/ijbs.120760 PMC12780948

[124] C. Hu , J. Yang , Z. Qi , et al., “Heat Shock Proteins: Biological Functions, Pathological Roles, and Therapeutic Opportunities,” MedComm 3, no. 3 (2022): e161. 35928554 10.1002/mco2.161 PMC9345296

[125] A. Parrales and T. Iwakuma , “p53 as a Regulator of Lipid Metabolism in Cancer,” International Journal of Molecular Sciences 17, no. 12 (2016): 2074. 27973397 10.3390/ijms17122074 PMC5187874

[126] L. Shen , X. Sun , Z. Fu , et al., “The Fundamental Role of the p53 Pathway in Tumor Metabolism and Its Implication in Tumor Therapy,” Clinical Cancer Research 18, no. 6 (2012): 1561–1567. 22307140 10.1158/1078-0432.CCR-11-3040

[127] N. G. Yoon , S. Y. Kim , S. Y. Jung , et al., “The Pro‐tumorigenic Functions of Cancer‐Associated Adipocytes Are Dependent on the Mitochondrial Chaperone Tumor Necrosis Factor Receptor‐Associated Protein 1,” Signal Transduction and Targeted Therapy 11, no. 1 (2026): 294. 42509229 10.1038/s41392-026-02861-8 PMC13408860

[128] K. Oliva , G. Barker , G. E. Rice , et al., “D‐DIGE to Identify Proteins Associated With Gestational Diabetes in Omental Adipose Tissue,” Journal of Endocrinology 218, no. 2 (2013): 165–178. 23709000 10.1530/JOE-13-0010

[129] P. M. Grierson , P. B. Dodhiawala , Y. Cheng , et al., “The MK2/Hsp27 Axis Is a Major Survival Mechanism for Pancreatic Ductal Adenocarcinoma Under Genotoxic Stress,” Science Translational Medicine 13, no. 622 (2021): eabb5445. 34851698 10.1126/scitranslmed.abb5445 PMC9475471

[130] I. Rzeszutek , M. Cybularczyk‐Cecotka , A. Deręgowska , et al., “New Mitochondria‐Targeted Fisetin Derivative Compromises Mitophagy and Limits Survival of Drug‐Induced Senescent Breast Cancer Cells,” Journal of Medicinal Chemistry 67, no. 19 (2024): 17676–17689. 39322603 10.1021/acs.jmedchem.4c01664 PMC11472315

[131] F. R. P. Dewi , S. Jiapaer , A. Kobayashi , et al., “Nucleoporin TPR (Translocated Promoter Region, Nuclear Basket Protein) Upregulation Alters MTOR‐HSF1 Trails and Suppresses Autophagy Induction in Ependymoma,” Autophagy 17, no. 4 (2021): 1001–1012. 32207633 10.1080/15548627.2020.1741318 PMC8078762

[132] J. Yi , J. Zhu , J. Wu , et al., “Oncogenic Activation of PI3K‐AKT‐mTOR Signaling Suppresses Ferroptosis via SREBP‐Mediated Lipogenesis,” PNAS 117, no. 49 (2020): 31189–31197. 33229547 10.1073/pnas.2017152117 PMC7733797

[133] H. R. Jin , J. Wang , Z. J. Wang , et al., “Lipid Metabolic Reprogramming in Tumor Microenvironment: From Mechanisms to Therapeutics,” Journal of Hematology & Oncology 16, no. 1 (2023): 103. 37700339 10.1186/s13045-023-01498-2 PMC10498649

[134] K. J. Park , R. B. Gaynor , and Y. T. Kwak , “Heat Shock Protein 27 Association With the I Kappa B Kinase Complex Regulates Tumor Necrosis Factor Alpha‐Induced NF‐kappa B Activation,” Journal of Biological Chemistry 278, no. 37 (2003): 35272–35278. 12829720 10.1074/jbc.M305095200

[135] R. Sur , P. A. Lyte , and M. D. Southall , “Hsp27 regulates Pro‐inflammatory Mediator Release in Keratinocytes by Modulating NF‐kappaB Signaling,” Journal of Investigative Dermatology 128, no. 5 (2008): 1116–1122. 18007587 10.1038/sj.jid.5701157

[136] H. F. Alkan , K. E. Walter , A. Luengo , et al., “Cytosolic Aspartate Availability Determines Cell Survival When Glutamine Is Limiting,” Cell Metabolism 28, no. 5 (2018): 706–720.e6. 30122555 10.1016/j.cmet.2018.07.021 PMC6390946

[137] J. Garcia‐Bermudez , L. Baudrier , K. La , et al., “Aspartate Is a Limiting Metabolite for Cancer Cell Proliferation Under Hypoxia and in Tumours,” Nature Cell Biology 20, no. 7 (2018): 775–781. 29941933 10.1038/s41556-018-0118-z PMC6030478

[138] A. Okazaki , P. A. Gameiro , D. Christodoulou , et al., “Glutaminase and Poly(ADP‐Ribose) Polymerase Inhibitors Suppress Pyrimidine Synthesis and VHL‐Deficient Renal Cancers,” Journal of Clinical Investigation 127, no. 5 (2017): 1631–1645. 28346230 10.1172/JCI87800 PMC5409089

[139] C. Sandoval‐Acuña , N. Torrealba , V. Tomkova , et al., “Targeting Mitochondrial Iron Metabolism Suppresses Tumor Growth and Metastasis by Inducing Mitochondrial Dysfunction and Mitophagy,” Cancer Research 81, no. 9 (2021): 2289–2303. 33685989 10.1158/0008-5472.CAN-20-1628

[140] A. Dey and R. P. Barnes , “Genetic Approaches for Targeted Oxidative Stress,” NAR cancer 7, no. 4 (2025): zcaf049. 41446758 10.1093/narcan/zcaf049 PMC12723233

[141] M. J. Iqbal , A. Kabeer , Z. Abbas , et al., “Interplay of Oxidative Stress, Cellular Communication and Signaling Pathways in Cancer,” Cell Communication and Signaling 22, no. 1 (2024): 7. 38167159 10.1186/s12964-023-01398-5 PMC10763046

[142] G. S. Shadel and T. L. Horvath , “Mitochondrial ROS Signaling in Organismal Homeostasis,” Cell 163, no. 3 (2015): 560–569. 26496603 10.1016/j.cell.2015.10.001 PMC4634671

[143] C. N. Okoye , S. A. Koren , and A. P. Wojtovich , “Mitochondrial Complex I ROS Production and Redox Signaling in Hypoxia,” Redox Biology 67 (2023): 102926. 37871533 10.1016/j.redox.2023.102926 PMC10598411

[144] H. Du , T. Xu , S. Yu , et al., “Mitochondrial Metabolism and Cancer Therapeutic Innovation,” Signal Transduction and Targeted Therapy 10, no. 1 (2025): 245. 40754534 10.1038/s41392-025-02311-x PMC12319113

[145] C. Cömert , K. Kjær‐Sørensen , J. Hansen , et al., “HSP60 Chaperone Deficiency Disrupts the Mitochondrial Matrix Proteome and Dysregulates Cholesterol Synthesis,” Molecular Metabolism 88 (2024): 102009. 39147275 10.1016/j.molmet.2024.102009 PMC11388177

[146] B. Texier , M. Prime , D. Atamena , et al., “Mortalin/Hspa9 Involvement and Therapeutic Perspective in Parkinson's Disease,” Neural Regeneration Research 18, no. 2 (2023): 293–298. 35900406 10.4103/1673-5374.346487 PMC9396523

[147] Y. Zheng , J. Sun , Z. Luo , et al., “Emerging Mechanisms of Lipid Peroxidation in Regulated Cell Death and Its Physiological Implications,” Cell Death & Disease 15, no. 11 (2024): 859. 39587094 10.1038/s41419-024-07244-x PMC11589755

[148] G. Shen , J. Liu , Y. Wang , et al., “Ferroptosis in Cancer and Inflammatory Diseases: Mechanisms and Therapeutic Implications,” MedComm 6, no. 9 (2025): e70349. 40919133 10.1002/mco2.70349 PMC12409078

[149] J. Szyller and I. Bil‐Lula , “Heat Shock Proteins in Oxidative Stress and Ischemia/Reperfusion Injury and Benefits From Physical Exercises: A Review to the Current Knowledge,” Oxidative Medicine and Cellular Longevity 2021 (2021): 6678457. 33603951 10.1155/2021/6678457 PMC7868165

[150] P. C. Ikwegbue , P. Masamba , B. E. Oyinloye , et al., “Roles of Heat Shock Proteins in Apoptosis, Oxidative Stress, Human Inflammatory Diseases, and Cancer,” Pharmaceuticals (Basel, Switzerland) 11, no. 1 (2017): 2. 29295496 10.3390/ph11010002 PMC5874698

[151] K. Guo , N. X. Kang , Y. Li , et al., “Regulation of HSP27 on NF‐kappaB Pathway Activation May be Involved in Metastatic Hepatocellular Carcinoma Cells Apoptosis,” BMC Cancer 9 (2009): 100. 19331697 10.1186/1471-2407-9-100 PMC2681475

[152] H. Zhang , W. Gong , S. Wu , et al., “Hsp70 in Redox Homeostasis,” Cells 11, no. 5 (2022): 829. 35269451 10.3390/cells11050829 PMC8909019

[153] V. Ngo and M. L. Duennwald , “Nrf2 and Oxidative Stress: A General Overview of Mechanisms and Implications in Human Disease,” Antioxidants (Basel, Switzerland) 11, no. 12 (2022): 2345. 36552553 10.3390/antiox11122345 PMC9774434

[154] X. Liang , J. Weng , Z. You , et al., “Oxidative Stress in Cancer: From Tumor and Microenvironment Remodeling to Therapeutic Frontiers,” Molecular Cancer 24, no. 1 (2025): 219. 40847302 10.1186/s12943-025-02375-x PMC12372290

[155] A. S. Chalova , M. V. Sudnitsyna , P. I. Semenyuk , et al., “Effect of Disulfide Crosslinking on Thermal Transitions and Chaperone‐Like Activity of human Small Heat Shock Protein HspB1,” Cell Stress & Chaperones 19, no. 6 (2014): 963–972. 24898092 10.1007/s12192-014-0520-9 PMC4389837

[156] X. Liu , K. Liu , C. Li , et al., “Heat‐shock Protein B1 Upholds the Cytoplasm Reduced state to Inhibit Activation of the Hippo Pathway in H9c2 Cells,” Journal of Cellular Physiology 234, no. 4 (2019): 5117–5133. 30256412 10.1002/jcp.27322

[157] T. Jung and T. Grune , “The Proteasome and the Degradation of Oxidized Proteins: Part I‐Structure of Proteasomes,” Redox Biology 1, no. 1 (2013): 178–182. 24024151 10.1016/j.redox.2013.01.004 PMC3757679

[158] E. R. Gallagher and E. L. F. Holzbaur , “The Selective Autophagy Adaptor p62/SQSTM1 Forms Phase Condensates Regulated by HSP27 That Facilitate the Clearance of Damaged Lysosomes via Lysophagy,” Cell Reports 42, no. 2 (2023): 112037. 36701233 10.1016/j.celrep.2023.112037 PMC10366342

[159] S. Guo , W. Wharton , P. Moseley , et al., “Heat Shock Protein 70 Regulates Cellular Redox Status by Modulating Glutathione‐Related Enzyme Activities,” Cell Stress & Chaperones 12, no. 3 (2007): 245–254. 17915557 10.1379/CSC-265.1 PMC1971240

[160] H. S. Li , Y. N. Zhou , L. Li , et al., “HIF‐1α Protects Against Oxidative Stress by Directly Targeting Mitochondria,” Redox Biology 25 (2019): 101109. 30686776 10.1016/j.redox.2019.101109 PMC6859547

[161] T. Bahr , J. Katuri , T. Liang , et al., “Mitochondrial Chaperones in Human Health and Disease,” Free Radical Biology & Medicine 179 (2022): 363–374. 34780988 10.1016/j.freeradbiomed.2021.11.015 PMC8893670

[162] C. Espinosa‐Diez , V. Miguel , D. Mennerich , et al., “Antioxidant Responses and Cellular Adjustments to Oxidative Stress,” Redox Biology 6 (2015): 183–197. 26233704 10.1016/j.redox.2015.07.008 PMC4534574

[163] J. F. Liu , P. C. Chen , T. Y. Ling , et al., “Hyperthermia Increases HSP Production in Human PDMCs by Stimulating ROS Formation, p38 MAPK and Akt Signaling, and Increasing HSF1 Activity,” Stem Cell Research & Therapy 13, no. 1 (2022): 236. 35659731 10.1186/s13287-022-02885-1 PMC9166587

[164] S. Akter , R. Madhuvilakku , A. K. Kar , et al., “Reactive Oxygen Species (ROS) in Cancer: From Mechanism to Therapeutic Implications,” Signal Transduction and Targeted Therapy 11, no. 1 (2026): 111. 41881983 10.1038/s41392-026-02583-x PMC13018652

[165] H. Jiang , J. Zuo , B. Li , et al., “Drug‐Induced Oxidative Stress in Cancer Treatments: Angel or Devil?,” Redox Biology 63 (2023): 102754. 37224697 10.1016/j.redox.2023.102754 PMC10220276

[166] H. Tang , J. Li , X. Liu , et al., “Down‐Regulation of HSP60 Suppresses the Proliferation of Glioblastoma Cells via the ROS/AMPK/mTOR Pathway,” Scientific Reports 6 (2016): 28388. 27325206 10.1038/srep28388 PMC4914999

[167] X. Zhang , Y. Fan , and K. Tan , “A Bird's Eye View of Mitochondrial Unfolded Protein Response in Cancer: Mechanisms, Progression and Further Applications,” Cell Death & Disease 15, no. 9 (2024): 667. 39261452 10.1038/s41419-024-07049-y PMC11390889

[168] R. Kumar , A. K. Chaudhary , J. Woytash , et al., “A Mitochondrial Unfolded Protein Response Inhibitor Suppresses Prostate Cancer Growth in Mice via HSP60,” Journal of Clinical Investigation 132, no. 13 (2022): e149906. 35653190 10.1172/JCI149906 PMC9246382

[169] J. R. Braxton , H. Shao , E. Tse , et al., “Asymmetric Apical Domain States of Mitochondrial Hsp60 Coordinate Substrate Engagement and Chaperonin Assembly,” Nature Structural & Molecular Biology 31, no. 12 (2024): 1848–1858. 10.1038/s41594-024-01352-0 PMC11638070 38951622

[170] R. Mitra , K. Wu , C. Lee , et al., “ATP‐Independent Chaperones,” Annual Review of Biophysics 51 (2022): 409–429. 10.1146/annurev-biophys-090121-082906 35167761

[171] M. Taipale , I. Krykbaeva , M. Koeva , et al., “Quantitative Analysis of HSP90‐Client Interactions Reveals Principles of Substrate Recognition,” Cell 150, no. 5 (2012): 987–1001. 22939624 10.1016/j.cell.2012.06.047 PMC3894786

[172] S. Modi , A. Stopeck , H. Linden , et al., “HSP90 Inhibition is Effective in Breast Cancer: A Phase II Trial of Tanespimycin (17‐AAG) plus Trastuzumab in Patients With HER2‐Positive Metastatic Breast Cancer Progressing on Trastuzumab,” Clinical Cancer Research 17, no. 15 (2011): 5132–5139. 21558407 10.1158/1078-0432.CCR-11-0072

[173] C. Peng , J. Brain , Y. Hu , et al., “Inhibition of Heat Shock Protein 90 Prolongs Survival of Mice With BCR‐ABL‐T315I‐Induced Leukemia and Suppresses Leukemic Stem Cells,” Blood 110, no. 2 (2007): 678–685. 17395781 10.1182/blood-2006-10-054098 PMC1924476

[174] G. S. Nowakowski , A. K. McCollum , M. M. Ames , et al., “A Phase I Trial of Twice‐weekly 17‐allylamino‐demethoxy‐geldanamycin in Patients With Advanced Cancer,” Clinical Cancer Research 12, no. 20 Pt 1 (2006): 6087–6093. 17062684 10.1158/1078-0432.CCR-06-1015

[175] A. Budina‐Kolomets , M. R. Webster , J. I. Leu , et al., “HSP70 Inhibition Limits FAK‐Dependent Invasion and Enhances the Response to Melanoma Treatment With BRAF Inhibitors,” Cancer Research 76, no. 9 (2016): 2720–2730. 26984758 10.1158/0008-5472.CAN-15-2137 PMC4939897

[176] L. Neckers and P. Workman , “Hsp90 Molecular Chaperone Inhibitors: Are We There Yet?,” Clinical Cancer Research 18, no. 1 (2012): 64–76. 22215907 10.1158/1078-0432.CCR-11-1000 PMC3252205

[177] M. V. Powers and P. Workman , “Targeting of Multiple Signalling Pathways by Heat Shock Protein 90 Molecular Chaperone Inhibitors,” Endocrine‐Related Cancer 13, no. Suppl 1 (2006): S125–135. 17259553 10.1677/erc.1.01324

[178] R. Garcia‐Carbonero , A. Carnero , and L. Paz‐Ares , “Inhibition of HSP90 Molecular Chaperones: Moving Into the Clinic,” Lancet Oncology 14, no. 9 (2013): e358–369. 23896275 10.1016/S1470-2045(13)70169-4

[179] H. J. Patel , S. Modi , G. Chiosis , et al., “Advances in the Discovery and Development of Heat‐Shock Protein 90 Inhibitors for Cancer Treatment,” Expert Opinion Drug Discovery 6, no. 5 (2011): 559–587. 10.1517/17460441.2011.563296 PMC3293194 22400044

[180] K. Jhaveri , S. O. Ochiana , M. P. Dunphy , et al., “Heat Shock Protein 90 Inhibitors in the Treatment of Cancer: Current Status and Future Directions,” Expert Opinion on Investigational Drugs 23, no. 5 (2014): 611–628. 24669860 10.1517/13543784.2014.902442 PMC4161020

[181] R. Bao , C. J. Lai , H. Qu , et al., “CUDC‐305, a Novel Synthetic HSP90 Inhibitor With Unique Pharmacologic Properties for Cancer Therapy,” Clinical Cancer Research 15, no. 12 (2009): 4046–4057. 19509149 10.1158/1078-0432.CCR-09-0152

[182] T. Taldone , A. Gozman , R. Maharaj , et al., “Targeting Hsp90: Small‐Molecule Inhibitors and Their Clinical Development,” Current Opinion in Pharmacology 8, no. 4 (2008): 370–374. 18644253 10.1016/j.coph.2008.06.015 PMC2760289

[183] Z. N. Li and Y. Luo , “HSP90 Inhibitors and Cancer: Prospects for Use in Targeted Therapies (Review),” Oncology Reports 49, no. 1 (2023): 6. 36367182 10.3892/or.2022.8443 PMC9685368

[184] G. Chiosis , C. S. Digwal , J. B. Trepel , et al., “Structural and Functional Complexity of HSP90 in Cellular Homeostasis and Disease,” Nature Reviews Molecular Cell Biology 24, no. 11 (2023): 797–815. 37524848 10.1038/s41580-023-00640-9 PMC10592246

[185] P. J. Gray Jr. , T. Prince , J. Cheng , et al., “Targeting the Oncogene and Kinome Chaperone CDC37,” Nature Reviews Cancer 8, no. 7 (2008): 491–495. 18511936 10.1038/nrc2420 PMC2779120

[186] S. Sreeramulu , S. L. Gande , M. Göbel , et al., “Molecular Mechanism of Inhibition of the human Protein Complex Hsp90‐Cdc37, a Kinome Chaperone‐Cochaperone, by Triterpene Celastrol,” Angewandte Chemie 48, no. 32 (2009): 5853–5855. 19585625 10.1002/anie.200900929

[187] M. A. Serwetnyk and B. S. J. Blagg , “The Disruption of Protein‐Protein Interactions With Co‐Chaperones and Client Substrates as a Strategy Towards Hsp90 Inhibition,” Acta Pharm Sin B 11, no. 6 (2021): 1446–1468. 34221862 10.1016/j.apsb.2020.11.015 PMC8245820

[188] R. Adão , L. M. Zanphorlin , T. B. Lima , et al., “Revealing the Interaction Mode of the Highly Flexible Sorghum Bicolor Hsp70/Hsp90 Organizing Protein (Hop): A Conserved Carboxylate Clamp Confers High Affinity Binding to Hsp90,” Journal of Proteomics 191 (2019): 191–201. 29425735 10.1016/j.jprot.2018.02.007

[189] S. M. Hoy , “Pimitespib: First Approval,” Drugs 82, no. 13 (2022): 1413–1418. 35986838 10.1007/s40265-022-01764-6

[190] Y. Kurokawa , Y. Honma , A. Sawaki , et al., “Pimitespib in Patients With Advanced Gastrointestinal Stromal Tumor (CHAPTER‐GIST‐301): A Randomized, Double‐Blind, Placebo‐Controlled Phase III Trial,” Annals of Oncology 33, no. 9 (2022): 959–967. 35688358 10.1016/j.annonc.2022.05.518

[191] E. S. Polson , V. B. Kuchler , C. Abbosh , et al., “KHS101 disrupts Energy Metabolism in Human Glioblastoma Cells and Reduces Tumor Growth in Mice,” Science Translational Medicine 10, no. 454 (2018): eaar2718. 30111643 10.1126/scitranslmed.aar2718

[192] G. Xu and J. Y. Li , “ATP5A1 and ATP5B Are Highly Expressed in Glioblastoma Tumor Cells and Endothelial Cells of Microvascular Proliferation,” Journal of Neuro‐Oncology 126, no. 3 (2016): 405–413. 26526033 10.1007/s11060-015-1984-x

[193] C. Caruso Bavisotto , G. Alberti , A. M. Vitale , et al., “Hsp60 Post‐Translational Modifications: Functional and Pathological Consequences,” Frontiers in Molecular Biosciences 7 (2020): 95. 32582761 10.3389/fmolb.2020.00095 PMC7289027

[194] S. Mulati , R. Jiang , J. Wang , et al., “6‐Shogaol Exhibits a Promoting Effect With Tax via Binding HSP60 in Non‐Small‐Cell Lung Cancer,” Cells 11, no. 22 (2022): 3678. 36429106 10.3390/cells11223678 PMC9688423

[195] K. Wiechmann , H. Müller , S. König , et al., “Mitochondrial Chaperonin HSP60 Is the Apoptosis‐Related Target for Myrtucommulone,” Cell Chemical Biology 24, no. 5 (2017): 614–623.e6. 28457707 10.1016/j.chembiol.2017.04.008

[196] S. Venkatesh and C. K. Suzuki , “HSP60 Takes a Hit: Inhibition of Mitochondrial Protein Folding,” Cell Chemical Biology 24, no. 5 (2017): 543–545. 28525768 10.1016/j.chembiol.2017.05.011

[197] Y. Sekiguchi , M. Yamada , T. Noguchi , et al., “The Anti‐Cancer Drug gefitinib Accelerates Fas‐Mediated Apoptosis by Enhancing Caspase‐8 Activation in Cancer Cells,” Journal of Toxicological Sciences 44, no. 6 (2019): 435–440. 31168030 10.2131/jts.44.435

[198] Z. Wang , T. Song , Z. Guo , et al., “A Novel Hsp70 Inhibitor Specifically Targeting the Cancer‐Related Hsp70‐Bim Protein‐Protein Interaction,” European Journal of Medicinal Chemistry 220 (2021): 113452. 33906046 10.1016/j.ejmech.2021.113452

[199] S. H. Park , K. H. Baek , I. Shin , et al., “Subcellular Hsp70 Inhibitors Promote Cancer Cell Death via Different Mechanisms,” Cellular and Chemical Biology 25, no. 10 (2018): 1242–1254.e8. 10.1016/j.chembiol.2018.06.010 30057298

[200] M. Q. Pham , T. H. V. Tran , Q. L. Pham , et al., “In Silico Analysis of the Binding Properties of Solasonine to Mortalin and p53, and in Vitro Pharmacological Studies of Its Apoptotic and Cytotoxic Effects on human HepG2 and Hep3b Hepatocellular Carcinoma Cells,” Fundamental and Clinical Pharmacology 33, no. 4 (2019): 385–396. 30628118 10.1111/fcp.12447

[201] Z. Balogi , G. Multhoff , T. K. Jensen , et al., “Hsp70 Interactions With Membrane Lipids Regulate Cellular Functions in Health and Disease,” Progress in Lipid Research 74 (2019): 18–30. 30710597 10.1016/j.plipres.2019.01.004

[202] L. Fiengo , G. Lauro , M. L. Bellone , et al., “The Plant Diterpene Epoxysiderol Targets Hsp70 in Cancer Cells, Affecting Its ATPase Activity and Reducing Its Translocation to Plasma Membrane,” International Journal of Biological Macromolecules 189 (2021): 262–270. 34437915 10.1016/j.ijbiomac.2021.08.138

[203] X. Qiu , X. Guan , W. Liu , et al., “DAL‐1 Attenuates Epithelial to Mesenchymal Transition and Metastasis by Suppressing HSPA5 Expression in Non‐Small Cell Lung Cancer,” Oncology Reports 38, no. 5 (2017): 3103–3113. 29048640 10.3892/or.2017.6000

[204] D. Nayak , A. Katoch , D. Sharma , et al., “Indolylkojyl Methane Analogue IKM5 Potentially Inhibits Invasion of Breast Cancer Cells via Attenuation of GRP78,” Breast Cancer Research and Treatment 177, no. 2 (2019): 307–323. 31175498 10.1007/s10549-019-05301-0

[205] S. Rastogi , A. Joshi , N. Sato , et al., “An Update on the Status of HSP90 Inhibitors in Cancer Clinical Trials,” Cell stress & chaperones 29, no. 4 (2024): 519–539. 38878853 10.1016/j.cstres.2024.05.005 PMC11260857

[206] A. Rajan , R. J. Kelly , J. B. Trepel , et al., “A Phase I Study of PF‐04929113 (SNX‐5422), an Orally Bioavailable Heat Shock Protein 90 Inhibitor, in Patients With Refractory Solid Tumor Malignancies and Lymphomas,” Clinical Cancer Research 17, no. 21 (2011): 6831–6839. 21908572 10.1158/1078-0432.CCR-11-0821 PMC3207004

[207] K. Jhaveri , K. Miller , L. Rosen , et al., “A Phase I Dose‐escalation Trial of Trastuzumab and Alvespimycin Hydrochloride (KOS‐1022; 17 DMAG) in the Treatment of Advanced Solid Tumors,” Clinical Cancer Research 18, no. 18 (2012): 5090–5098. 22781552 10.1158/1078-0432.CCR-11-3200

[208] C. Peng , F. Zhao , H. Li , et al., “HSP90 Mediates the Connection of Multiple Programmed Cell Death in Diseases,” Cell Death & Disease 13, no. 11 (2022): 929. 36335088 10.1038/s41419-022-05373-9 PMC9637177

[209] R. Kalluri and V. S. LeBleu , “The Biology, Function, and Biomedical Applications of Exosomes,” Science (New York, NY) 367, no. 6478 (2020): eaau6977. 10.1126/science.aau6977 PMC7717626 32029601

[210] S. M. Batool , T. Hsia , A. Beecroft , et al., “Extrinsic and Intrinsic Preanalytical Variables Affecting Liquid Biopsy in Cancer,” Cell Reports Medicine 4, no. 10 (2023): 101196. 37725979 10.1016/j.xcrm.2023.101196 PMC10591035

[211] S. Tukaj , “Dual Role of Autoantibodies to Heat Shock Proteins in Autoimmune Diseases,” Frontiers in Immunology 15 (2024): 1421528. 38903496 10.3389/fimmu.2024.1421528 PMC11187000

[212] B. Hu , G. Liu , K. Zhao , et al., “Diversity of Extracellular HSP70 in Cancer: Advancing From a Molecular Biomarker to a Novel Therapeutic Target,” Frontiers in Oncology 14 (2024): 1388999. 38646439 10.3389/fonc.2024.1388999 PMC11026673

[213] X. Wang , J. Huang , W. Chen , et al., “The Updated Role of Exosomal Proteins in the Diagnosis, Prognosis, and Treatment of Cancer,” Experimental & Molecular Medicine 54, no. 9 (2022): 1390–1400. 36138197 10.1038/s12276-022-00855-4 PMC9535014

[214] M. T. Hsu , Y. K. Wang , and Y. J. Tseng , “Exosomal Proteins and Lipids as Potential Biomarkers for Lung Cancer Diagnosis, Prognosis, and Treatment,” Cancers 14, no. 3 (2022): 732. 35158999 10.3390/cancers14030732 PMC8833740

[215] J. A. Welsh , D. C. I. Goberdhan , L. O'Driscoll , et al., “Minimal Information for Studies of Extracellular Vesicles (MISEV2023): From Basic to Advanced Approaches,” Journal of Extracellular Vesicles 13, no. 2 (2024): e12404. 38326288 10.1002/jev2.12404 PMC10850029

[216] C. Caruso Bavisotto , F. Cappello , A. J. L. Macario , et al., “Exosomal HSP60: A Potentially Useful Biomarker for Diagnosis, Assessing Prognosis, and Monitoring Response to Treatment,” Expert Review of Molecular Diagnostics 17, no. 9 (2017): 815–822. 28718351 10.1080/14737159.2017.1356230

[217] A. Hoshino , H. S. Kim , L. Bojmar , et al., “Extracellular Vesicle and Particle Biomarkers Define Multiple Human Cancers,” Cell 182, no. 4 (2020): 1044–1061.e18. 32795414 10.1016/j.cell.2020.07.009 PMC7522766

[218] X. Wang , R. Ni , X. Wang , et al., “Targeting HSP90 in Cancer: Advances in the Development of Inhibitors, Mechanisms of Action, and Therapeutic Applications,” Molecular Cancer 25, no. 1 (2026): 102. 41792781 10.1186/s12943-025-02559-5 PMC13081375

[219] X. Liang , R. Chen , C. Wang , et al., “Targeting HSP90 for Cancer Therapy: Current Progress and Emerging Prospects,” Journal of Medicinal Chemistry 67, no. 18 (2024): 15968–15995. 39256986 10.1021/acs.jmedchem.4c00966

[220] J. Oberoi , X. A. Guiu , E. A. Outwin , et al., “HSP90‐CDC37‐PP5 Forms a Structural Platform for Kinase Dephosphorylation,” Nature Communications 13, no. 1 (2022): 7343. 10.1038/s41467-022-35143-2 PMC9709061 36446791

[221] R. Kalluri and K. M. McAndrews , “The Role of Extracellular Vesicles in Cancer,” Cell 186, no. 8 (2023): 1610–1626. 37059067 10.1016/j.cell.2023.03.010 PMC10484374

[222] A. Kawazoe , K. Itahashi , N. Yamamoto , et al., “TAS‐116 (Pimitespib), an Oral HSP90 Inhibitor, in Combination With Nivolumab in Patients With Colorectal Cancer and Other Solid Tumors: An Open‐Label, Dose‐Finding, and Expansion Phase Ib Trial (EPOC1704),” Clinical Cancer Research 27, no. 24 (2021): 6709–6715. 34593531 10.1158/1078-0432.CCR-21-1929

[223] M. Halilovic , M. Abdelsalam , J. Zabkiewicz , et al., “Selective Degradation of Mutant FMS‐Like Tyrosine Kinase‐3 Requires BIM‐Dependent Depletion of Heat Shock Proteins,” Leukemia 38, no. 12 (2024): 2561–2572. 39300221 10.1038/s41375-024-02405-5 PMC11588663

[224] F. H. Schopf , M. M. Biebl , and J. Buchner , “The HSP90 Chaperone Machinery,” Nature Reviews Molecular Cell Biology 18, no. 6 (2017): 345–360. 28429788 10.1038/nrm.2017.20

[225] F. Gentile , J. C. Yaacoub , J. Gleave , et al., “Artificial Intelligence‐Enabled Virtual Screening of Ultra‐Large Chemical Libraries With Deep Docking,” Nature Protocols 17, no. 3 (2022): 672–697. 35121854 10.1038/s41596-021-00659-2

[226] S. García‐Alonso , P. Mesa , L. P. Ovejero , et al., “Structure of the RAF1‐HSP90‐CDC37 Complex Reveals the Basis of RAF1 Regulation,” Molecular Cell 82, no. 18 (2022): 3438–3452.e8. 36055235 10.1016/j.molcel.2022.08.012

[227] Y. Gomez‐Llorente , F. Jebara , M. Patra , et al., “Structural Basis for Active Single and Double Ring Complexes in Human Mitochondrial Hsp60‐Hsp10 Chaperonin,” Nature Communications 11, no. 1 (2020): 1916. 10.1038/s41467-020-15698-8 PMC7174398 32317635

[228] S. Stangl , M. Gehrmann , J. Riegger , et al., “Targeting Membrane Heat‐Shock Protein 70 (Hsp70) on Tumors by cmHsp70.1 Antibody,” Proceedings of the National Academy of Sciences of the United States of America 108, no. 2 (2011): 733–738. 21187371 10.1073/pnas.1016065108 PMC3021082

[229] C. S. Fan , H. C. Hung , C. C. Chen , et al., “Development of a Humanized Antibody Targeting Extracellular HSP90α to Suppress Endothelial‐Mesenchymal Transition‐Enhanced Tumor Growth of Pancreatic Adenocarcinoma Cells,” Cells 13, no. 13 (2024): 1146. 38994997 10.3390/cells13131146 PMC11240389

[230] L. Seclì , L. Avalle , P. Poggio , et al., “Targeting the Extracellular HSP90 Co‐Chaperone Morgana Inhibits Cancer Cell Migration and Promotes Anticancer Immunity,” Cancer Research 81, no. 18 (2021): 4794–4807. 34193441 10.1158/0008-5472.CAN-20-3150

[231] P. Xu , J. C. Yang , S. Ning , et al., “Allosteric Inhibition of HSP70 in Collaboration With STUB1 Augments Enzalutamide Efficacy in Antiandrogen Resistant Prostate Tumor and Patient‐Derived Models,” Pharmacological Research 189 (2023): 106692. 36773708 10.1016/j.phrs.2023.106692 PMC10162009

[232] G. Zhao , M. Zhu , Y. Li , et al., “Using DNA‐Encoded Libraries of Fragments for Hit Discovery of Challenging Therapeutic Targets,” Expert Opinion on Drug Discovery 19, no. 6 (2024): 725–740. 38753553 10.1080/17460441.2024.2354287

[233] A. M. Ray , N. Salim , M. Stevens , et al., “Exploiting the HSP60/10 Chaperonin System as a Chemotherapeutic Target for Colorectal Cancer,” Bioorganic & Medicinal Chemistry 40 (2021): 116129. 33971488 10.1016/j.bmc.2021.116129 PMC8194340

[234] O. Bloch , C. A. Crane , Y. Fuks , et al., “Heat‐shock Protein Peptide Complex‐96 Vaccination for Recurrent Glioblastoma: A Phase II, Single‐Arm Trial,” Neuro‐oncology 16, no. 2 (2014): 274–279. 24335700 10.1093/neuonc/not203 PMC3895386

[235] N. Ji , Y. Zhang , Y. Liu , et al., “Heat Shock Protein Peptide Complex‐96 Vaccination for Newly Diagnosed Glioblastoma: A Phase I, Single‐arm Trial,” JCI Insight 3, no. 10 (2018): e99145. 29769450 10.1172/jci.insight.99145 PMC6012501

[236] N. Hebbar , R. Epperly , A. Vaidya , et al., “CAR T Cells Redirected to Cell Surface GRP78 Display Robust Anti‐Acute Myeloid Leukemia Activity and Do Not Target Hematopoietic Progenitor Cells,” Nature Communications 13, no. 1 (2022): 587. 10.1038/s41467-022-28243-6 PMC8803836 35102167

[237] J. Ibanez , N. Hebbar , U. Thanekar , et al., “GRP78‐CAR T Cell Effector Function Against Solid and Brain Tumors Is Controlled by GRP78 Expression on T Cells,” Cell Reports Medicine 4, no. 11 (2023): 101297. 37992682 10.1016/j.xcrm.2023.101297 PMC10694756

[238] A. Bashiri Dezfouli , M. Yazdi , M. R. Benmebarek , et al., “CAR T Cells Targeting Membrane‐Bound Hsp70 on Tumor Cells Mimic Hsp70‐Primed NK Cells,” Frontiers in Immunology 13 (2022): 883694. 35720311 10.3389/fimmu.2022.883694 PMC9198541

[239] Z. Albakova and Y. Mangasarova , “The HSP Immune Network in Cancer,” Frontiers in Immunology 12 (2021): 796493. 34917098 10.3389/fimmu.2021.796493 PMC8669653

[240] C. Wood , P. Srivastava , R. Bukowski , et al., “An Adjuvant Autologous Therapeutic Vaccine (HSPPC‐96; Vitespen) versus Observation Alone for Patients at High Risk of Recurrence After Nephrectomy for Renal Cell Carcinoma: A Multicentre, Open‐Label, Randomised Phase III Trial,” Lancet (London, England) 372, no. 9633 (2008): 145–154. 18602688 10.1016/S0140-6736(08)60697-2

[241] K. Misawa , H. Bhat , P. S. Adusumilli , et al., “Combinational CAR T‐Cell Therapy for Solid Tumors: Requisites, Rationales, and Trials,” Pharmacology & Therapeutics 266 (2025): 108763. 39617146 10.1016/j.pharmthera.2024.108763 PMC11848936

[242] A. Ianevski , K. Nader , K. Driva , et al., “Single‐cell Transcriptomes Identify Patient‐Tailored Therapies for Selective Co‐Inhibition of Cancer Clones,” Nature Communications 15, no. 1 (2024): 8579. 10.1038/s41467-024-52980-5 PMC11450203 39362905

[243] J. Lim , V. Chin , K. Fairfax , et al., “Transitioning Single‐Cell Genomics Into the Clinic,” Nature Reviews Genetics 24, no. 8 (2023): 573–584. 10.1038/s41576-023-00613-w 37258725

